# Advancements in Passive Wireless Sensors, Materials, Devices, and Applications

**DOI:** 10.3390/s23198200

**Published:** 2023-09-30

**Authors:** Denghui He, Yuanhui Cui, Fangchao Ming, Weiping Wu

**Affiliations:** 1 School of Information Science and Engineering, Dalian Polytechnic University, Dalian 116034, China; 220510811000485@xy.dlpu.edu.cn (D.H.); 210520854000590@xy.dlpu.edu.cn (F.M.); 2 Laboratory of Thin Film Optics, Shanghai Institute of Optics and Fine Mechanics, Chinese Academy of Sciences, 390 Qinghe Road, Jiading District, Shanghai 201800, China; 3 Key Laboratory of Materials for High Power Laser, Shanghai Institute of Optics and Fine Mechanics, Chinese Academy of Sciences, 390 Qinghe Road, Jiading District, Shanghai 201800, China

**Keywords:** wireless sensors, nanogenerators, self-powered wireless sensors, sensor networks

## Abstract

In recent years, passive wireless sensors have been studied for various infrastructure sectors, making them a research and development focus. While substantial evidence already supports their viability, further effort is needed to understand their dependability and applicability. As a result, issues related to the theory and implementation of wireless sensors still need to be resolved. This paper aims to review and summarize the progress of the different materials used in different passive sensors, the current status of the passive wireless sensor readout devices, and the latest peripheral devices. It will also cover other related aspects such as the system equipment of passive wireless sensors and the nanogenerators for the energy harvesting for self-powered sensors for applications in contemporary life scenarios. At the same time, the challenges for future developments and applications of passive wireless are discussed.

## 1. Introduction

As new sciences progress, technologies in industry areas also rapidly develop over time. An important part of this is how to increase the level of automation at the industrial level. It has become a challenge to achieve a faster and more accurate representation of analogue quantities when reading data [1,2,3]. The rapid development of detection systems in various fields has promoted various sensing technologies, but the layout and deployment of wired sensors have become their main drawbacks, such as power supplies and batteries occupying a large volume of space and the difficulties of post-maintenance management, greatly limiting the performance of sensor systems [4,5,6]. The frequent replacement of the battery can also cause an increase in economic costs that cannot recycle the battery material in the sensor in a timely manner [7]. The leakage of battery material from sensors that are not disposed of promptly can also lead to the contamination of land and water [8]. Some sensors are difficult to take out easily when they are put into use in the environment [9]. In such environmental requirements, the use of active devices can cause a lot of damage to the enclosed environment itself, whereas wireless sensors allow for a single input and long-term or even permanent use. Especially in some extreme cases of high temperature and pressure, integrated circuits and most active electronics do not work properly, while passive wireless sensors can still complete functional characteristics without active devices, and self-powered sensors can avoid battery replacement and the real-time monitoring of targets [10,11,12]. The advent of wireless sensors can realize the monitoring of closed environments, such as the monitoring of the human internal environmental health index, the monitoring of drugs and food in vacuum packaging, and the monitoring of building safety structures in dangerous areas [13,14,15,16].

Passive wireless sensors are mainly composed of passive sensing units and external readout systems that can achieve both passive resonance and wireless sensing. Resonance means that the sensor can work without a power supply and wireless sensing means that the sensor’s detection information can be obtained through wireless sensing. The passive wireless sensor sensing mechanism is divided into optical and electrical in two ways [17]. This paper mainly describes the wireless sensing implemented by electrical means. The difference in the sensor is mainly manifested in two forms, mainly in the difference in the manner of energy transmission and the modulation of the signal. In terms of energy transmission, one type is applied to the far field area of the electromagnetic field, which is called a radiation field sensor, and the other type is mainly applied to the near field range of the electromagnetic field, which is called an induction field sensor. To meet the distance requirements of the radiation field, the antenna used is very large, so it is difficult to apply to small systems. Inductive field sensors rely on inductive coupling and can be used in small spaces [18]. In terms of signal processing, it is divided into RFID type, harmonic type, and passive resonance type. In addition, the self-powered sensor can also be understood as a narrow passive wireless sensor because it does not require an external power supply. Currently, passive wireless sensor technology has drawn a lot of attention. With the rapid development of Micro electro mechanical systems (MEMS) technology and IC technology, passive wireless sensors have improved greatly in terms of size and power consumption [19,20]. Nanosensors are considered ideal for powering wireless sensors, and a wide variety of nanogenerators have made the use of wireless sensors even more widespread [21,22,23]. These operating characteristics make passive wireless sensors and self-powered sensors have the advantages of long life and flexibility for many application scenarios. Therefore, the research on passive wireless sensors has great potential for a broad range of applications.

This review paper is divided into six parts. The first part mainly introduces the main classification of passive wireless sensors at present, as well as the working principles of various types, to be clearer about the focus of sensor development. The second part focuses on the sensitive materials for passive wireless sensors and introduces the various types of sensors made of novel materials for different applications. In the third part, the update of the passive wireless sensor reader is introduced, in addition to some peripheral devices such as built-in switches and card readers. The fourth part starts with how to make the nano-energy harvesting device more efficient, then describes the development of energy harvesting algorithms and nanomaterials. It also includes improvements in the use of energy equipment. The fifth part summarizes the application status of passive wireless sensors and self-powered sensors in daily life based on the first four parts. The sixth part summarizes the article, describes the defects of the existing technology, and puts forward the direction and prospect of the future development of passive wireless sensors.

## 2. Passive Wireless Sensor Classification and Principle

To understand passive wireless sensors more clearly and quickly, its classification and working principle are the first subjects to be introduced. By further understanding the working principle of each type of sensor, we can provide a more convenient choice for practical applications.

### 2.1. RFID Sensor

In response to the requirement that passive wireless sensors can only be used in a narrow area, RFID sensors are miniaturized sensor networks that combine radio frequency identification technology (RFID) tag antennas with wireless sensor network technology (WSN) [24]. The main role of WSN is to obtain environmental information or send the internal information of the supervised object, and the role of RFID is to identify and monitor the monitored object [25,26]. RFID is a low-cost technology that enables passive wireless data communication. To reduce the size of the sensor network, RF energy is a sustainable and reliable energy source; using RF to charge sensors can not only extend the life of nodes, but also calculate and sense at anytime, anywhere by combining passive marker antennas with a variety of sensors. An RFID sensor is also known as a Backscattering modulation sensor, which is a combination of RF identification technology and MEMS sensor technology. Firstly, the sensing system sends the carrier signal to the sensing system, and then realizes the wireless output of the signal through the modulation and demodulation of the sensor information. A complete RFID sensor system includes a readout system with a transmitting antenna and several sensor units [27]. By reading out the antenna, the RF signal can be sent to the sensor receiving antenna, and the signal energy can be rectified and stabilized, so that the RFID chip and the sensor can work. After being modulated by the sensitive unit, it backscatters back and is picked up by the readout antenna. These energy signals contain unique corresponding information, which can sense information such as pressure and temperature [28]. RFID can operate in different frequency bands. The low frequency (LF) (30~300 kHz) and high frequency (HF) (3~30 MHz) bands use magnetic flux coupling, and the ultra-high frequency (UHF) (300 MHz~3 GHz) band uses electromagnetic wave coupling. A UHF frequency band communication distance >16 m is better than LF and HF frequency bands [29]. There are two ways to modulate the signal: frequency modulation or phase modulation. The invention utilizes RFID technology to combine sensitive information with RFID signals, so as to achieve the purpose of wireless reading. RFID-type sensors have gradually enabled the remote identification of wirelessly readable, writable, and passive electronic tags. RFID-type sensors do not use batteries, but because the processing and sensitive modulation of RF signals still need to use complex active circuits, RFID-type sensors can be classified as passive wireless sensors in a broad sense [30].

### 2.2. Harmonic Scattering Sensor

This kind of sensor modulates the input signal of the antenna through the frequency doubling and mixing effect of nonlinear devices such as diodes, thus converting the sensing information into frequency information and scattering it to the readout antenna [31]. Then, using the traditional test instrument or modern digital processing technology, these signals are analyzed and calculated, and the frequency, amplitude, and phase of harmonic components are obtained [32]. The sensing system usually includes a sensing tag and a reader. A wireless signal is sent, received, and evaluated by a card reader, and at the same time, the tag produces a backscatter effect [33]. These tags are completely passive and have almost no chips. Under normal circumstances, the harmonic scattering sensor can be divided into two types; they mainly use the diode frequency multiplier effect generated by the high order harmonics to load the sensor information, at this time, because the sensor receives signal and transmits signal in two frequency bands, so whether it is the readout end or the sensor end, you need two antennas to send and receive the signal, respectively [34]. The second type is based on the semiconductor “mixing” effect; the two adjacent frequencies are sent to the detector through the detector, and then through the detector modulation and semiconductor mixing, to obtain a “difference frequency” output, and then it is fed back to the detector, so that the detector only needs one antenna to work [35]. Due to the need to use diodes, and as these diodes are only used as passive nonlinear components, in a broad sense such harmonic scattering sensors can also be referred to as passive wireless sensors.

### 2.3. Self-Powered Sensor

Energy harvesting sensors do not contain traditional batteries, but are usually equipped with an energy capture unit, such as piezoelectric nanogenerators (PiENG), thermoelectric nanogenerators (PyENG), friction nanogenerators (TENG) [36], etc., which can collect the tiny energy ubiquitous in the working environment and convert it into electricity, thereby driving the normal work of the sensor. This is called Self-Powered technology. According to the different objects of energy acquisition, energy acquisition can be divided into mechanical energy, thermal energy, chemical energy, and electromagnetic energy. The collected electric energy can be supplied to the sensor and processor after rectification, filtering, voltage regulation, and other processing. This kind of sensor, in the basic structure of the active wireless sensor node is not essentially different, but with an energy collection unit instead of the battery, its circuit is more complex, and it also needs to use active devices such as diodes and transistors, but it does not need an external power supply, so it can also be classified as a generalized passive wireless sensor. Therefore, this is a self-powered sensor using a new type of energy harvesting device.

PiENG is a kind of device that converts external mechanical energy into electrical energy by using nano-scale piezoelectric body as an energy source. At present, there are several vibration energy acquisition technologies based on electromagnetic drive, electrostatic drive, and magneto strictive drive at home and abroad [37]. However, the existing energy acquisition technology is difficult to meet the needs of micro sensors, and the electromagnetic energy acquisition technology has some problems, such as the size of the magnet and coil being too large, the output voltage being too low, and it being easy to be affected by electromagnetic interference. Electrostatic requirements are that there must be an external power supply or a power supply that has a small allowable gap and a high output impedance. The scalable structure with delay has obvious nonlinearity and a complex structure. Therefore, PiENG, with its small size, no electromagnetic interference, simple structure, and other advantages, has more development prospects. During the operation of the wind power generation system, mechanical energy such as wind, sound wave/ultrasonic wave, noise, and mechanical vibration causes the deformation of piezoelectric materials. According to the physical properties of piezoelectric ceramics, when squeezed or twisted by external forces, the distribution of positive and negative charges inside will change, forming a potential difference in both directions. By connecting this potential difference to a line, it can be converted into a usable form of electricity. In general, piezoelectric nanogenerators are composed of piezoelectric materials, conductive substrates, metal electrodes, etc. [38], thereby converting the mechanical energy into electrical energy [39]. The PiENG can be roughly divided into two kinds: one is to apply external force to the axis of the nanowires, so that the nanowires are squeezed and stretched on one side and the other is to cause uniaxial compression of the nanowires when the external force is parallel to the axial direction of the nanowires, thus forming a potential difference.

PyENG mainly use the temperature difference existing in nature, such as the temperature difference between the skin and the outside world during skin repair. The thermoelectric conversion technology we commonly use is achieved through the Seebeck effect, although the Seebeck effect can provide more energy, but there are certain limitations, that is, the need for two different substances to form a loop and the temperature difference between the two substances being very large, and in a large range, to achieve the output of electrical energy [40]. Therefore, PyENG usually takes advantage of the pyroelectric effect of nano-pyroelectric materials to convert excess thermal energy into electrical energy for microsensors. The working principle of pyroelectric nanogenerators can be divided into two kinds: One is the primary pyroelectric effect, which is the process of changing the polarization intensity of pyroelectric materials caused by temperature changes, and then generating electric charges. The secondary pyroelectric effect refers to the deformation of the material due to thermal expansion, which changes the polarization intensity of the material through the piezoelectric effect, so that it generates electric charge [41]. Due to the use of green semiconductor materials, based on the pyroelectric effect of the working principle, the research and application of nanoscale thermoelectric power generation systems is very broad.

TENG is a new type of device based on triboelectric and electrostatic induction [42]. At present, the technical equipment using triboelectrification is mainly triboelectrification, although the electricity generated can reach tens of thousands of volts, but its current is very small, usually only a few microamps. Therefore, its practical application value is not high [43]. Compared with friction power generation, its output current is much larger, so it has more application prospects. The basic principle is that two different polymers are coated or coated with a layer of metal at one end to form a new type of nanopower generation system. The two polymers need to be very different in their electron trapping capacity, and there needs to be a gap between the two polymers. It is formed by the friction between two polymer layers under the action of one or more external forces. The separation of charges forms a potential, thereby realizing the effect of tribomechanical energy collection, whose energy output depends on the expansion of the interface’s microscopic contact area and the subsequent increase in interface electrons in the cloud overlap, resulting in a greater charge transfer [44,45].

### 2.4. Passive Resonance Type

The passive resonant sensor is essentially a resonant body, and its characteristic parameters, such as resonance frequency and quality factor, change with the change in the measured parameters [46,47,48]. The electromagnetic wave signal is sent to the sensor by an external receiving antenna, and its resonance frequency is excited, so that its resonance frequency can reach the target of wireless detection. The process generally uses an electromagnetic exciter to convert electrical energy into magnetic energy, and then the resonator vibrates mechanically, at which time the electromagnetic pickup begins to obtain resonant signals to convert mechanical energy into electrical energy. In such a cycle, the parameter can be detected. Depending on the resonant element, common resonance sensors include a vibrating string sensor, a vibrating cylinder sensor, a vibrating beam sensor, a vibrating film sensor, quartz crystal microbalance (QCM) [49], and a surface acoustic wave sensor (SAW) [50]. SAW sensors use the electrical signals of surface acoustic waves as they travel to detect environmental changes. QCM sensors use the vibration frequency of quartz crystals to detect environmental changes. In general, QCM sensors have high stability, and SAW sensors have high sensitivity [51]. According to its specific resonance theory, passive resonance sensors can be further divided into the LC resonator, the RF cavity type, the Patch Antenna type, the Surface Acoustic Wave (SAW) type, the Magnetoelastic resonator, etc. [52,53,54]. An LC passive wireless sensor usually adopts the structure of a flat spiral inductor and sensitive capacitor and realizes the integration of the two through micro-machining technology, and has a very small volume. The SAW and magnetoelastic resonant requires special materials, and the RF resonant and patch antenna resonance frequency is very high, up to GHz, which is more demanding for reading equipment. The structure of this passive sensor is very simple, without active components such as diodes and transistors. Therefore, it is a passive wireless sensor in a narrow sense.

## 3. Passive Wireless Sensor Manufacturing Materials and Manufacturing Technology

With the continuous development of applied chemistry and materials science, researchers have started exploring various materials as the main sensitive components for passive wireless sensors in different working environments. The aim is to enhance people’s quality of life and increase the efficiency of daily activities. There is a growing demand for sensor devices in many areas of life, including agriculture, industrial process control, and electronic processing. However, the high energy consumption, low accuracy, and difficult operation of many sensors have posed serious challenges to people’s lives under certain conditions, prompting increased attention in recent years.

This part mainly introduces the non-self-powered sensor type material selection and production process. The sensitive material is the core component of a sensor, and the sensitivity of the sensor largely depends on the sensitive material used. Conventional sensitive materials based on semiconductors and polymer materials each have their own advantages and disadvantages. With their high-quality properties, these sensitive materials are increasingly being used in different types of sensors, such as temperature sensors, humidity sensors, pressure sensors, hazardous gas sensors, chemical detection sensors, and so on.

### 3.1. Temperature Sensors

Gas insulated switchgear (GIS) are commonly used, and are widely used in power systems [55,56]. In 2015, Ma et al. proposed the design of a precise SAW temperature sensor. To prevent potential incomplete discharges in the sensor, an epoxy encapsulation and a ring antenna were employed. The use of this material in the sensor enabled it to be widely used in high voltage safety circuit systems to detect temperature issues in real-time. Experiments were conducted to test the accuracy and response time of the SAW sensor, and the feasibility of the scheme was proven [57]. In the same year, Chen Li et al. also contributed to the circuit temperature detection of high-voltage safety systems. The fixed inductor and variable capacitor circuit were embedded in alumina ceramic substrate by using high temperature-resistant material combined with embedded structure design. Experiments show that the sensor can perform real-time detection at room temperature to 1000 °C [58].

**Figure 1:**
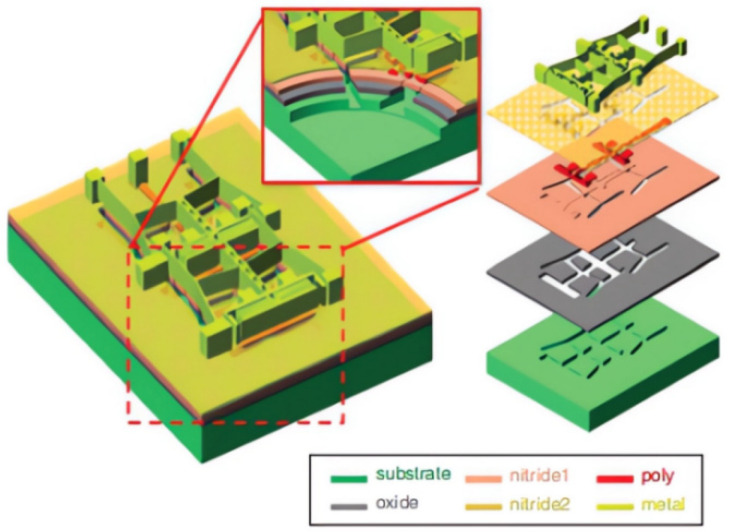
Simplified layout of a capacitive temperature sensor [61].

The Multi-User MEMS process system (MUMPs) process is commonly used in the production of microelectronic devices due to its ability to improve performance and facilitate mass production through standard processes. Currently, the MUMPs process is primarily used in the manufacturing of optical devices, gyroscopes, sensors, and other similar devices [59,60]. In 2016, Wang Lifeng and Huang Qing’an conducted further research on LC passive wireless sensors using the metal MUMPs process and the electroplated nickel micromachining process. They designed and fabricated a parallel capacitor that uses both the nickel metal layer and polysilicon layer as the electrode part of the temperature sensing capacitor. The silicon nitride layer and air gap were utilized as the isolation layer (Figure 1). The sensor demonstrated a mean sensitivity of 32.45 fF/°C over the temperature range of 0~100 °C and provided 46.61 kHz/°C over the full temperature range, making it a highly effective form of LC passive wireless sensor [61].

**Figure 2:**
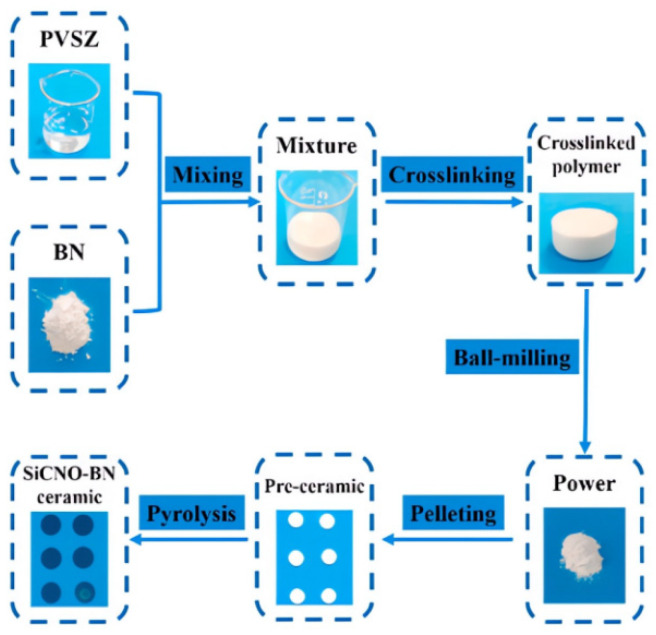
Fabrication process of SiCNO-BN ceramic discs [64].

Hexagonal boron nitride alloy ceramics exhibit exceptional properties such as high temperature resistance, thermal shock resistance, ablation resistance, dielectric transmission, resistance to molten metal erosion, and processability, making them suitable for use in high-temperature conditions [62,63]. In 2021, Yuxi Yu et al. synthesized SiCNO-BN composite ceramics using polyethylenesiloxane and hexagonal boron nitride, as shown in Figure 2, and utilized this material to develop a passive wireless temperature sensor. Experimental tests demonstrated that the SiCNO-BN temperature sensor can measure temperatures up to 1250 °C when doped with 10 wt to 20 wt BN content, which is higher than the 900 °C limit of the SiCNO temperature sensor. The incorporation of this new material is a significant breakthrough in temperature measurement technology [64].

**Figure 3:**
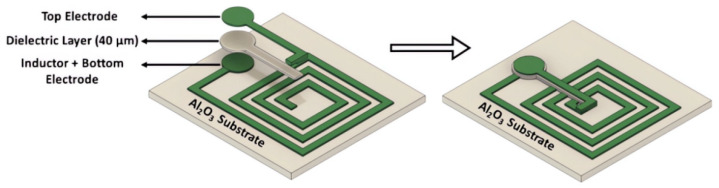
LC resonator structure [65].

In 2022, several studies showed that SAW sensors may not be suitable for practical temperature detection in high voltage safety circuit systems, as they have only been tested in laboratory scale experiments. As an alternative, Kavin Sivaneri et al. proposed the use of thermally stable and highly conductive ceramic oxides to construct LC resonators, using conductive ceramic materials instead of traditional fabrication methods involving metal electrodes deposited on a dielectric ceramic substrate. This process is illustrated in Figure 3. The ceramic material can only be fabricated through a high-temperature process, and the flat capacitors and inductors are printed on an Al_2_O_3_ substrate using a screen-printing technique. The sensors produced using this process were tested and found to have high sensitivity even in harsh environments [65].

Temperature has become a ubiquitous factor in everyday testing, and it even affects the transport of energy and the movement of molecules. The aforementioned examples provide new options for sensitive materials that can be used for passive wireless temperature sensors in specific applications.

### 3.2. Humidity Sensors

Graphene has a unique nanostructure and excellent electrical properties, while graphene oxide (GO) has a substantial number of oxygen-containing functional groups on its surface, which can provide reaction sites for chemical reactions [66,67]. In 2015, Zhang Cong and Wang Lifeng designed an LC passive wireless moisture sensor that works by the resonance frequency of the inductor being associated with the sensing capacitance, which varies with humidity. This project aimed to design a new kind of complementary metal oxide semiconductor (CMOS) intermediate capacitive humidity sensor chip on FR-4 substrate (FR-4), and to prepare a fixed planar spiral type copper inductor on this basis. The inductor–capacitor (LC) slot is formed by linking an adjustable capacitor to a fixed inductor with wires. The sensor uses GO instead of polyimide [68] as the sensing component. Practical tests have proven that GO can greatly enhance the sensitivity of the sensor, with a sensitivity of 18.75 kHz/%RH in the range of 15–95% RH [69]. In 2020, Liu Bo et al. exploited the diverse binding capabilities of different oxygen-containing functional groups to the target gas molecules and the fact that GO has a substantial number of functional groups. By adjusting the functional groups in the structure of GO, a selective humidity sensor can be designed to achieve a selective response to the target gas. After testing at 32.8% RH, the sensitivity of this GO was up to 40.1%, and the reaction and recovery curves remained largely consistent [70].

Silicon dioxide is chemically stable, so it can be used as a preparation sensor. However, the solubility of silicon dioxide is very poor, which does not facilitate the homogeneous dispersion of hydrophilic materials, posing a challenge for making moisture sensors using silicon dioxide [71]. In 2018, H. Zhao et al. utilized the mercapto-alkene click reaction of thiol-functionalized microporous silica nanoparticles (MSNs) to synthesize organic–inorganic hybrid materials that can be extracted in solutions. The best hybrid materials had a small particle size (105 nm), hydrophilic properties, and dispersion stability in water at a concentration of 50 mg/mL and were used to make sensitive films. Thus, humidity sensors employing the solution method are possible [72].

Humidity is a variable parameter in our surroundings and has a significant effect on numerous fields, such as agriculture, the electronics industry, warehousing, and medicine. Various types of metal oxides, polymeric synthetics, and electrolytes have become sensitive materials for sensors. These detectors are widely used and have good sensitivity, and these materials are proposed as a supplementary reference for the selection of sensitive materials for humidity sensors in the future.

### 3.3. Pressure Sensors

Passive pressure sensors usually use a sealed electric feed capacitance [73]. Girish Chitnis et al. used an oil-based ferrofluid, polyimide-embedded planar coil, and 25 μm thick polyimide film to make the pressure sensor easier to produce. The applied pressure can be well correlated with the nonlinear model of resonance frequency variation using experimental detection [74]. Sapphire crystals have a unique crystal configuration, good stress resistance, and the ability to withstand high temperatures, making them essential in the production of various components [75,76]. In 2017, John E. Rogers and Yong-Kyu Yoon designed a platinum capacitive component-based pressure sensor for microelectromechanical systems using sapphire, which has high thermal conductivity, high melting temperature, and high resistivity physical properties at high temperatures. The sensor can operate at temperatures above 1000 °C and uses the borfloloat^®^ 33 handle principle for static and dynamic pressure testing, which includes silicon membranes and aluminum capacitors, with a sensitivity of up to 5.1 kHz/Pa and a pressure linearity of up to 900 Pa. Based on this, a dynamic pressure sensor with a sensitivity of 21.7 kHz/Pa and a pressure linearity of 800 Pa has been developed [77].

The MEMS process has made it easier to fabricate vulnerable chips due to the diffusion, etching, oxidation, and metal sputtering processes involved [78,79,80]. In 2020, Chen Jing and Wu Tian fabricated antenna-integrated film bulk acoustic resonator (FBAR) devices using the MEMS process. These devices can operate at the resonance of the FBAR device, can receive and transmit electromagnetic waves through magnetoelectric effects, and can detect pressure changes in the ambient environment by wirelessly measuring the frequency change in the sensor. The measurement results show that the sensor has a pressure sensitivity of 1.5 ppm/kPa, and a linearity of 0.997 over a pressure range of 17 kPa to 200 kPa at 25 °C [81].

Sensors are widely used in industrial production and everyday life, and passive wireless pressure sensors will be used in various industrial environments, such as water conservancy, hydropower, construction, and assembly line production. Ceramic pressure sensors, strain gauge sensors, diffusion silicon pressure sensors, and sapphire pressure sensors are widely used in the market today, and different types of pressure sensors are used in different environments.

### 3.4. Gas Sensors

Polyvinylidene fluoride (PVDF) is a ferroelectric polymeric material used in various electronic devices such as sensors and memories [82,83,84]. In 2017, Zhang, M. et al. used (PVDF-HFP) polymer as a sensing material for capacitive CO_2_ sensor applications, which addressed the drawbacks of using conventional metal sensors with high operational temperatures and high power consumption by observing the variation in polymer dielectric constantly [85,86].

**Figure 4:**
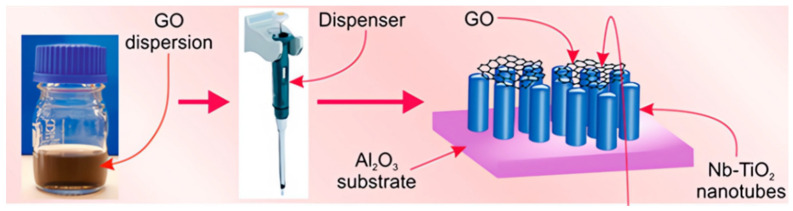
Schematic diagram of the fabrication process of the RGO/Nb-TiO_2_ hybrid material [89].

Environmental monitoring requires the ability to monitor low concentrations of toxic gases for air quality indicators. Regarding NO_2_ harmful gases, Mingsheng Ma et al. used two-dimensional tin disulfide (SnS_2_) nanostructures as materials to prepare gas-sensitive films using the casting method. A gas sensor based on low temperature co-fired ceramic technology was built [87]. In 2019, N. Van et al. synthesized a nanofiltration (NFs) sensor based on ZnFe_2_O_4_ (ZFO) using an on-chip electrostatic spinning technique and tested it in diverse annealing temperature ranges. The sensor mainly utilized the principle of a heterojunction between Reduced graphene oxide (RGO) and ZFO and the NFs consisted of many nanoparticles, thus forming a porous structure with a potential barrier at the grain boundaries, to obtain an enhanced sensor response. The best sensor response to H_2_S gas was acquired experimentally, and it was concluded that the best response to H_2_S gas was obtained by calcining a 1.0 wt% RGO-loaded ZFO-NF hybrid material sensor at 600 °C. The gas sensing results demonstrate a higher response than the pure ZFO sensor under the same conditions for the selected gas concentration and operating temperature range [88]. In addition, V Galstyan and A. Ponzoni developed hybrid structures using GO and niobium-doped titanium dioxide nanotubes (NT), which can detect hydrogen concentrations by correctly controlling the selectivity of the RGO sensor response. Based on this, a gas sensor with higher performance was developed by using graphene material and introducing a suitable niobium blend. The experimental data show that the sensor has a significantly enhanced response to hydrogen [89]. Figure 4 shows the preparation process.

With the increasing demand for renewable energy in modern-day life, ensuring security requirements in industrial processes has become crucial. Precisely detecting flammable and explosive gases and vapors remains a significant challenge. Therefore, the development of high-performance gas sensor devices has become a top priority. The accurate use of passive wireless gas sensors can help avoid the threat to human life from hazardous gases. The materials mentioned above are expected to be widely used in the development of new wireless passive gas sensors.

### 3.5. Biosensors

Phenylboronic acid (PBA) is a substance with high specificity to glucose, making it a promising material for glucose sensors. PBA is also easy to store and has high durability [90]. In 2020, Manik Dautta and Muhannad Alshetaiwi developed a PBA-hydrogel that responds to changes in ambient glucose concentration, resulting in significant shifts in the resonant response of the interlayer RF sensor. By coupling the interlayer RF resonator with the high dielectric constant of the PBA hydrogel, they were able to create electrically small glucose biosensors that functioned in the sub-band [91].

**Figure 5:**
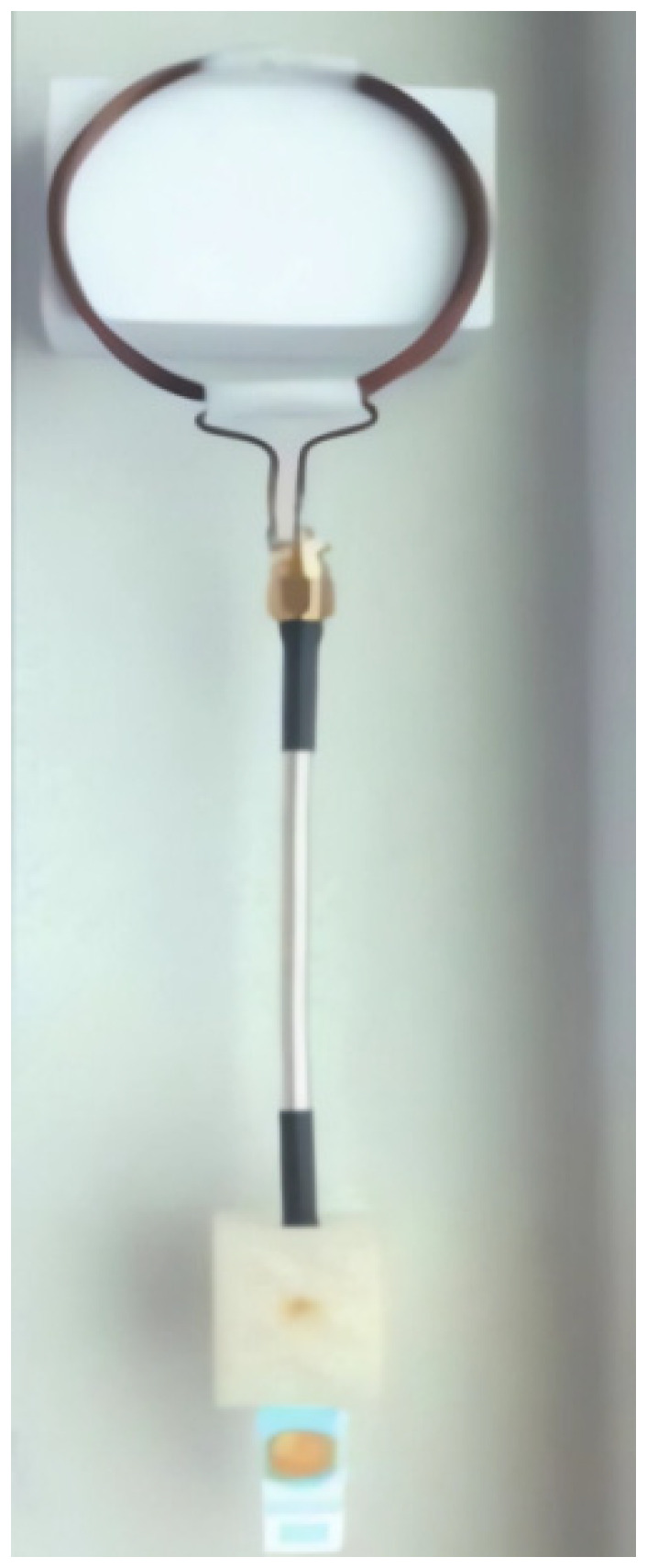
Prototype of the proposed wireless protease sensor [92].

The cross-linked gelatin-octanoic acid alloy possesses excellent stability, surface adhesion, and resistivity, and is a low-cost material. It is also protein degradable, making it an ideal candidate for wireless sensors for protein detection. Palraj Kalimuthu et al. developed a highly stable, but degradable in aqueous media, thermal gelatin-glycerol-CA composite for the wireless monitoring of proteolytic activities, such as tracking wounds and urinary tract infections. The proteolytic degradation of the composite can be used as a transduction mechanism for proteolytic activity in these wireless responders. The composite, consisting of cross-linked gelatin and octanoic acid, has great potential for applications that benefit from the wireless monitoring of protein hydrolysis activity, as illustrated in Figure 5 [92].

As technology continues to advance, and people become more health-conscious, there is an increasing demand for passive wireless biosensors that can measure various parameters of the human body in real-time. This represents a significant breakthrough in the medical field, as well as a reference for maintaining a healthy lifestyle. These passive wireless sensors, made from sensitive materials, have broad applications in chemical, biological, and medical fields (Table 1). There are the following restrictions on the selection of wireless passive sensor materials in real life applications. First of all, the energy absorption rate and conversion rate of the material are key performances, which will determine whether the wireless passive sensor can work stably. Second, because the wireless passive sensor itself is small in size, and the requirements for communication distance are different in different environments. At this time, to ensure the reliability of wireless transmission signals, some materials with low transmission loss and radiation loss need to be selected for material selection. These materials’ dielectric constant, permeability, and so on will affect wireless communication. Finally, when the sensor is used. External interference is a big problem to overcome, so it is necessary to constantly look for materials with high selectivity and shielding from external noise. For these performance requirements, it is necessary to use materials with low dielectric loss and low permeability, such as polymer materials mentioned in the paper and various composite polymer materials, to achieve efficient energy conversion. The size of the sensor also needs to be reasonably designed, and different sizes of materials will lead to different electromagnetic wave transmissions. Nano-structured materials can be used and doped with different proportions of chemical materials to try to achieve better results. For the removal of interference, some shielding layer materials can be added to the material preparation process to optimize the passive wireless sensor.

## 4. Passive Wireless Sensor Types and Optimization

Over the past decade, material preparation technology has evolved rapidly and introduced a variety of new passive wireless sensors into our daily lives. With this progress, to ultimately ensure the reliable transmission of data, the update of the reader has also put forward higher requirements. At present, wireless passive sensors still face problems such as detection distance and reading accuracy in wireless communication technology. Common communication technologies like ZigBee, Bluetooth, and wireless LAN can be used to receive commands and transmit data, but they also have substantial drawbacks such as high power consumption and large equipment, complex operation, and low flexibility. As a result, several studies have been conducted to address these issues. Standard passive wireless sensors include resonant circuit sensors, RFID-type sensors, SAW-type sensors, harmonic sensors, and intermodulation sensors. Furthermore, passive wireless sensors can be classified into passive low frequency (LF) sensors, high frequency (HF) sensors, and ultra-high frequency (UHF) sensors depending on their operating frequency. For different sensor types, algorithms are optimized accordingly.

Next, this part mainly introduces the innovation of wireless sensor reading methods, as well as the update of readers, and the breakthrough of peripheral devices. The main purpose of these contents is to improve the reliability of information transmission.

**Figure 6:**
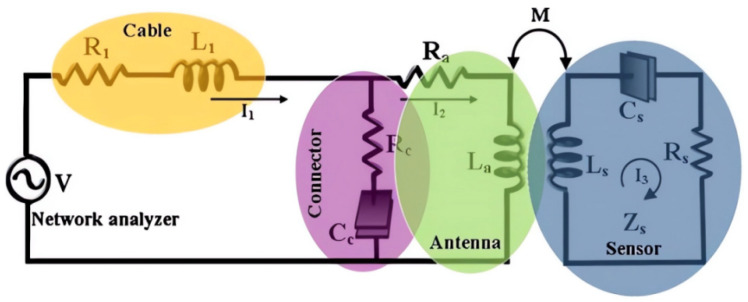
Wireless strain monitoring schematic [94].

Resonant circuit sensors are commonly used for measuring AC electric fields emitted from high-voltage equipment. In 2016, Mana Yazdani and Douglas J. Thomson proposed a passive wireless sensor that uses a coaxial transmission line resonant cavity, whose resonance frequency is disturbed by a capacitively coupled varistor. They used a sampling FPGA to interrogate the system and facilitate rapid sampling of the measured electric field, enhancing the AC field sampling rate. They also designed the sensor structure through finite component simulation and verified the feasibility of remote interrogation through measurement results [53,93]. Another study by Akbar Alipour, Emre Unal, and Sayim Gokyar proposed and developed a new passive RF resonator scheme with a specific architecture that allows for strong inductive coupling. This results in a higher wireless signal-to-noise ratio and better suppression of interference caused by noise. The ultra-thin multilayer (metal–dielectric–metal) structure used in the sensor obtains a linear relationship between the sensor frequency and the impedance pattern at different strain levels. The strain is derived from the slope of the response, reflecting good strain sensing performance with strong linearity [94,95,96,97]. The proposed system is illustrated in Figure 6.

Passive RFID tagging technology has many advantages over traditional bar code technology, including the ability to transmit information without requiring optical line-of-sight and the ability to read multiple tags simultaneously. However, designing and developing passive antenna sensors and systems that can perform both communication and sensing functions is a challenging task. One of the primary issues is the trade-off between resolution, sensitivity, size, read range, and robustness. To address these issues, Zhang Jun and Tian Guiyun summarized the solutions to improve wireless sensing distance by using passive RFID antennas and developing new sensing and communication strategies.

**Figure 7:**
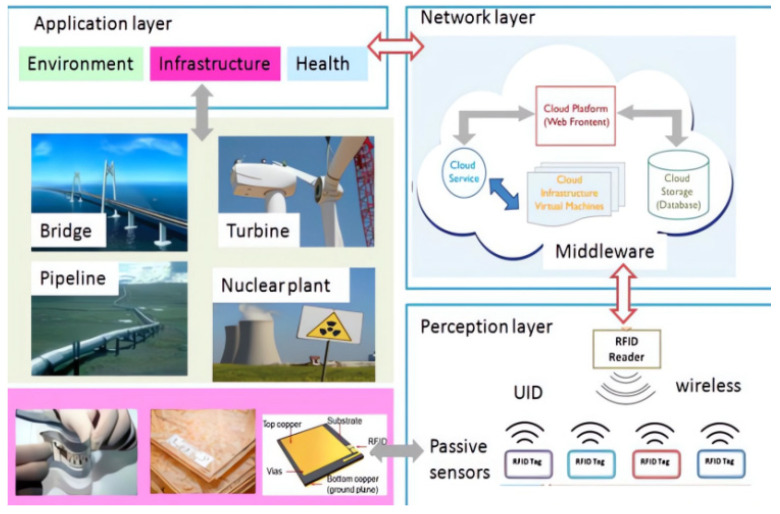
Passive RFID sensor network for SHM [99].

In addition, research is being conducted to address issues related to defect types, antenna sensors, measurement strategies, and feature extraction. For example, metal structure health monitoring (SHM) networks are being developed to detect defects, and new techniques are being developed to improve the adaptability and reliability of passive wireless sensors and systems (Figure 7). Overall, there is a need for continued research and innovation in this field to improve the accuracy, reliability, and functionality of passive wireless sensors and systems [97,98,99].

Real-time temperature monitoring is crucial in industrial production activities, particularly in the inspection of metal structures for defects. The wireless passive temperature sensor supported by SAW-RFID is well-suited to this scenario due to the potential alteration of physical and geometrical properties of the material, which can cause significant interference with the signal. With a long readout range and the ability to detect temperatures up to 250 °C, it offers a reliable solution. However, to address the issue of non-uniformity of different materials. Ali Imam Sunny and Jun Zhang propose a low-cost self-compensation method for self-sweeping measurements. This method involves selecting and fusing temperature-dependent features near the resonance region of the label based on passive low-frequency signals, demonstrating the possibility of bridging the gap between nondestructive and adaptive detection [100].

However, the general SAW-RFID is susceptible to environmental influences, leading to significant errors in measurement results. Tang Z. et al. proposed the multi-iterative enhanced two-point simple moving average method (MI-2P-SMA) to reduce the sensing error of the WP-SAW reflective delay line water temperature and pressure sensors. This method effectively reduces noise interference and significantly upgrades the sensing performance. The improved MI-2P-SMA method results in smaller comparative errors with increasing iteration time, offering an improved solution for environmental interference [101].

Given the growing population density, it is imperative to emphasize the importance of process monitoring and process effectiveness. Bernd Kubina and Christian Mandel have developed a passive wireless harmonic sensor system. This system utilizes chipless RF backscattering technology to encode and transmit measurement data, transforming the fundamental frequency signal into harmonics using a passive non-linear factor. This project introduces a novel type of amorphous chip sensor that consists of three main components, a receiving patch antenna, a harmonic generator, and a resonant antenna for transmission. The chip antenna receives an RF signal from a reader with a base frequency of “h” and connects the signal to a harmonic signal. This source can be encoded and transmitted by leveraging the temperature dependency of the dielectric constant of the DRA. By employing the harmonic backscattering of radio emissions, the sensor effectively eliminates interference from radar clutter [102].

Overall, both low and high frequency tags have a read range of up to 1 m, whereas UHF detectors offer a significantly greater read range in free air compared with low and high frequencies. When integrating sensors with remote power systems, the key factor to maximize wireless range is designing for low power consumption. In 2015, Shuangming Yu and Peng Feng addressed this concern by optimizing power consumption in passive wireless sensors through the utilization of the Epc Generation-2 UHF communication protocol. The sensor chip was fabricated using 0.18 µm CMOS technology, allowing for efficient space utilization. Specifically, the core chip of the sensor incorporates an on-chip temperature sensor and sensor interface, resulting in not only low power consumption but also reduced space wastage [103].

To enhance the transmission efficiency of wireless passive sensors, Dan Tang, Lifeng Wang, and their colleagues proposed a master-slave system for passive wireless multi-parameter sensors. This system enables simultaneous information transmission and power coupling by utilizing a coupling transformer for power transmission and receiving modulated data. The coupling transformer consists of a master-slave inductor. The secondary measurement system incorporates capacitive sensors for temperature, pressure, relative humidity, and the frequent conversion of capacitors is achieved through a relaxation oscillator. The system incorporates time-division multiplexing, allowing for the simultaneous measurement of multiple data sets. It is suitable for closed environments and utilizes the envelope method to recover the carrier signal. The pressure sensitivity is 5.6 fF/hPa, the temperature sensitivity is 250 fF/°C, and the relative humidity sensitivity is 71.4 fF/hPa [104].

In wireless power transmission systems, the coupling coefficient plays a crucial role in determining system performance. In the case of critical coupling, the coupling coefficient ensures that the output power of the load reaches its maximum. However, once this point is surpassed, the output power begins to decrease. Modifying the capacitance of the resonator in the critical coupling state allows for adjustment of the maximum output power. How to maximize the energy efficiency of the system under the premise of ensuring the maximum load is a very challenging subject. To address this, Dong Wook Lee, Jae Yun Lim, and others proposed a method of varying the resonance frequency of the resonator. By employing an over-coupled double-coil system, comparable performance to key coupling systems based on output power, voltage gain, and system energy efficiency can be achieved [105].

Haixia Zhang and Jingping Qiao introduced a new two-phase protocol that aims to enable efficient energy transfer and transmission. This protocol maximizes the utilization of existing relay equipment, specifically antenna equipment, while enhancing the safety performance of the system. The relay station incorporates a self-recovery of energy (S-ER) mechanism, effectively managing loopback interference data resulting from full-duplex (FD) operation. Additionally, it performs information relaying and achieves simultaneous radio power and information transmission in FD mode [106]. Furthermore, the utilization of antenna multiplexing for information collection significantly enhances the performance of the worst-case secrecy system.

In 2017, Mehdi Hajizadegan and Maryam Sakhdari made a significant discovery regarding the dual functionality of chemical sensing and RF modulation at the molecular level. This discovery brings forth numerous benefits, including reduced complexity and cost in low interference measurement implementations, as well as improved sensing linearity. By receiving the fundamental frequency signal and retransmitting it as a high frequency signal, this approach effectively detects both the average and fluctuations in chemical doping levels. The exceptional properties of graphene, such as high chemical sensitivity and frequency modulation capability in graphene field-effect transistors, allow for the development of nanomaterial-based harmonic transponder sensors. These sensors achieve low noise and low interference detection, even in challenging conditions characterized by extensive scattering and noise [107].

In 2018, Anish Babu and Boby George developed a readout scheme for LC sensors based on the impulse response principle. They introduced a simple and efficient method in which the readout coil performs a fast Fourier transform of the generated oscillating current. This approach enables the calculation of the system’s equivalent impedance at various frequencies. The resonance frequency of the coupled sensor coil is determined by identifying the maximum value of the imaginary part of this impedance. The quality factor Q of the sensor is measured using this approach. The feasibility of the method is demonstrated by minimizing the impact of noise on the output parameters through Gaussian curve fitting [95]. Marco Demori et al. proposed a contactless inquiry electronic technology that allows work to be performed within a few centimeters of the inquiry distance. The readout system measures the resonance frequency of the LC network by using a distance-independent time gating technique in a way that ensures that the readout frequency is of first order [108].

The advantages of remote measurement and the absence of power requirements have generated significant interest in passive wireless sensors. However, conventional sensors are typically limited to measuring a single parameter, which restricts their potential benefits. There is often a need for the simultaneous detection of multiple data parameters, presenting a challenge in achieving multi-parameter measurements with a single sensor. To tackle this issue, Wenjun Deng and Lifeng Wang propose a symmetrical LC topology circuit configuration comprising two LC resonators. Through a time-division multiplexed approach, dual-parameter monitoring is achieved. The symmetric LC structure enables the separation of resonance frequencies of distinct LC resonant cavities, allowing for simultaneous monitoring of two parameters. Experimental results validate the effectiveness of this configuration for dual-parameter monitoring. The symmetric LC topology circuit configuration, utilizing multiple LC resonators, emerges as a universal approach for multiparameter monitoring [109].

**Figure 8:**
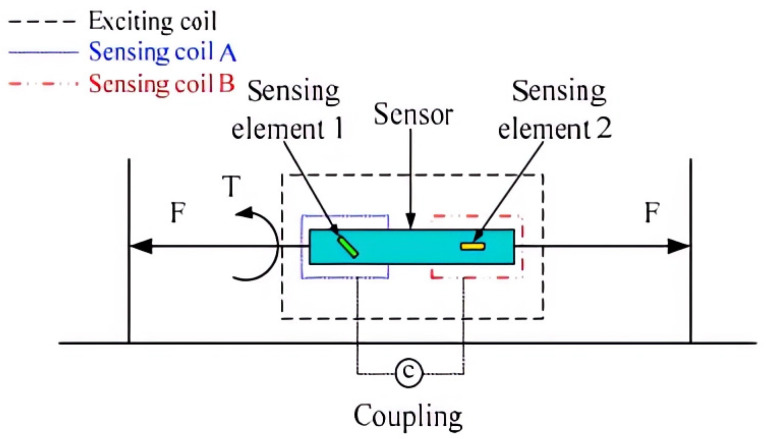
Schematic diagram of the operating principle [111].

In order to overcome the limitations of passive wireless sensors with a single output parameter, Zhang Yu and Hu Yanjun propose a data acquisition and transmission system that utilizes passive sensors and multi-parameter hybrid packaging technology. This system incorporates three main host computers, employs time counting sampling based on STC (system-on-chip) 89c52, and utilizes a wireless ad hoc network transmission based on the ZigBee protocol stack [110]. Simultaneously, the system places stringent accuracy requirements on the sensor. To enhance measurement accuracy, a two-dimensional approach is employed for measuring the same parameter. Yisong Tan, Jianhua Zhu, and Limin Ren introduce a novel two-dimensional passive pressure monitoring sensor principle, depicted in Figure 8. This principle addresses the challenge of conventional sensors needing to transmit data and power directly through an electronic circuit, which is unsuitable for the real-time monitoring of internal stresses in structures over extended periods. However, this method is limited to one-dimensional force detection and cannot discern two-dimensional force [111].

Optimizing passive wireless sensors involves more than just updating the sensor nodes themselves. Advancements in peripheral devices can also contribute to significant improvements in sensor performance. In 2019, Wang Lifeng and Wang Jinquan proposed a passive wireless switch array based on MEMS switches. They employed inductive coupling to enable the use of AC signal loading and frequency as power and control signals. Through tests, they confirmed the selective switching of the AC control signal to the switching branches, allowing for the extension of branch quality as needed. In addition, we can design into integrated circuits, thus greatly reducing the circuit size. This switching system finds application in controlling the multifunctional sensor or actuator arrays within a sealed space [112].

In 2020, Colm Mc Caffrey and Jacek Flak introduced a zero-power inductive network probing reader. They utilized a special frequency scanning algorithm to enhance the frequency resolution of the reader, resulting in an improved readout resolution for capacitive sensors in the tag. This approach eliminated the requirement for the sensor node to transmit data to the reader, as the reader could directly retrieve data from the sensor. The maximum finite distance for reader modulation was optimized, and signal isolation techniques were employed to enhance the accuracy of sensor reading by reducing the impact of stray capacitance [113].

In 2021, Naor Zohar shifted from receiving data transmitted by wireless sensor networks (WSNs) over wireless sensor networks to utilizing external devices (readers) for direct data reading during transmission. The polling rate of passive sensors was analyzed by collecting initial data at specific time points to determine the minimum monitoring rate required for accurate data collection. The operation of the WSN was accomplished through the collection of this initial data [114].

**Figure 9:**
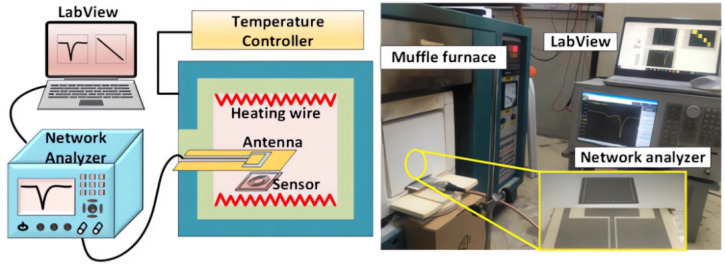
Schematic diagram of the high temperature test rig [115].

In 2022, Hairong Kou and colleagues introduced a wireless passive temperature sensor that relies on a complementary open-loop resonator (CSRR). The sensor’s parameters were fine-tuned using HFSS simulation software to enable the operation of a passive CSRR-integrated substrate integrated waveguide (SIW) resonator within the 2.5 GHz frequency band. Through the optimization of sensing techniques and leveraging the microwave backscattering principle, this sensor enables multi-point temperature testing in high-temperature environments, as depicted in Figure 9. Additionally, it allows for multi-parameter testing capabilities [115].

The third part first introduces methods to improve the efficiency and robustness of the information transmission of sensors such as resonant circuit sensors, RFID sensors, and SAW sensors, and then proposes updates on readers and peripheral devices, which all provide great contributions to information transmission. Table 2 summarizes the advantages and disadvantages of each of these typical types of passive wireless sensors.

## 5. Self-Powered Wireless Sensor Energy Harvesting Devices

Energy harvesting is a method of converting surrounding energy sources into electricity. Common sources of energy include solar, kinetic, electromagnetic, thermal, RF signals, microwaves, and ambient magnetic fields. As science and artificial intelligence advance, the development of intelligent small devices and their integration into emerging Internet of Things (IoT) applications, such as supply chain management and wearable computing, becomes crucial for meeting energy requirements. While small devices consume minimal power, the use of batteries can lead to significant environmental pollution, and the use of generators can result in increased space usage and reduced device performance. To address these challenges, small energy harvesting devices have been introduced and are being employed in wireless sensors. Wireless sensors offer two inherent advantages. Firstly, they eliminate the need for batteries, which reduces the sensors’ size and footprint, enabling a wider range of applications and facilitating the use of miniaturized devices. Secondly, the lifespan of wireless sensors is not limited by battery capacity but rather relies on the materials of the sensor itself, resulting in significantly extended service life.

**Figure 10:**
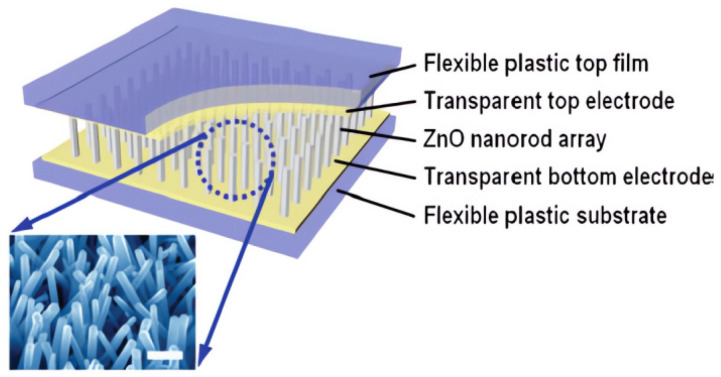
Schematic diagram of a flexible energy harvester in early use [116,117].

However, effectively harnessing the energy harvesting of wireless sensors brings its own challenges. The amount of energy available depends on the energy harvesting algorithm used and the nanomaterials used for energy loading, for example, the flexible energy collectors used in the past. Figure 10 shows the preparation of ZnO nanorods on a flexible plastic substrate using an aqueous solution method. This production method is easy to operate, has a low growth temperature, is suitable for large-scale growth and mass production, and has now evolved from this early research on the piezoelectric output of single-strain ZnO nanorods to innovations in nanorod arrays, flexible substrates, alternative materials, and various nanostructures [116,117].

This chapter focuses on methods to improve the efficiency of energy harvesting and advances in nanostructured generators that absorb different energy sources. Also included are new nanomaterials for TENG, PyENG, PiENG, and beneficial innovations for applications in various nanogenerators.

### 5.1. Algorithms

The key to energy collection is not only nanomaterials; novel energy collection algorithms can reduce energy waste and improve conversion efficiency. Maria Gorlatova and John Sarik et al. propose an IoT-based wireless node for harvesting kinetic (motion) energy. They address the energy distribution problem by introducing an optimal off-line algorithm, efficient approximation model, and optimal online algorithm case. The energy gained is calculated using trajectories, including acceleration trajectories, and the analysis and application of these trajectories enable the calculation of harvested energy. By focusing on the impact of energy characteristics on the adaptive harvesting algorithm, they implement a motion-driven continuous node within an ultra-low power architecture [118].

Energy-harvesting sensor networks (EHSNs) play a crucial role in long-term, large-scale data collection and are essential for supporting emerging technologies like big data and the Internet of Things. However, the random and dynamic nature of energy harvesting systems presents significant challenges in terms of energy consumption and replenishment. Additionally, to avoid conflicts with cellular users, sensors often need to suspend signaling and yield to other devices. In this context, the resource scheduling problem in EHSNs becomes particularly challenging. To address these challenges, Deyu Zhang and Zhigang Chen propose an efficiency optimization method based on aggregated networks specifically designed for EHSNs. Their approach offers an efficient and convenient online resource scheduling algorithm that effectively captures and optimizes disordered energy collection and consumption in the system. The method employs Lyapunov optimization to break down the problem into three manageable subcomponents, battery management, sample rate control, and data rate and channel configuration. By determining the optimal solution interval and providing boundaries for the optimal solution, this method ensures optimal aggregation network effectiveness. It also considers guaranteed energy and time constraints on the nodes, achieving optimal time-averaged aggregation network utility [119].

WSNs have limited power resources and need to operate for extended periods once deployed. To enhance the longevity and sustainability of WSN nodes, it is crucial to reduce their energy consumption and improve energy harvesting efficiency. As a node’s energy level decreases, the accuracy and quality of the generated sensor data also decline. Several common solutions are employed to address this issue, including communication power management, voltage and current regulation, power modes, duty cycles, and data fusion. Additionally, adaptive frequency usage is commonly employed in WSNs to minimize energy consumption.

Another approach to reducing energy consumption is employing adaptive sampling techniques, where the sampling rate is dynamically adjusted based on observed phenomena. By aligning the sampling rate with the occurrence of events, the energy-intensive sampling process is avoided during periods of low activity. However, adaptive sampling alone only extends the node’s lifecycle without ensuring long-term sustainability. Bruno Srbinovski and Michele Magno propose an adaptive sampling technique based on energy sensitivity, which is applied to energy management. This algorithm enhances the energy sensing capability of wireless sensing nodes, particularly those equipped with energy harvesting capabilities that rely on external sources for timed charging. The method ensures sustainable node operation even in scenarios with high energy consumption or when the collected energy is insufficient to sustain normal operation. When the collected energy falls below the energy supply threshold, any of the adaptive sampling rates can be utilized to maintain normal operation below the threshold [120].

Improving energy efficiency is crucial for maximizing the lifetime of WSNs, making the rational and efficient use of power supply capacity a key consideration. One significant challenge in achieving this is the time-varying and spatial dependence on renewable energy sources, which restricts access to these energy forms. The level of ambient energy available is closely tied to the current environmental conditions and exhibits significant variations. However, the dynamic nature and availability of environmental energy present new challenges.

To tackle this issue, S. Kosunalp proposes a solar energy forecasting algorithm called QLSEP, which is based on q-learning. This algorithm utilizes previous observations to predict future meteorological conditions, specifically focusing on the changing intensity of sunshine during solar energy collection. Building upon this forecasting capability, a novel energy-saving strategy is introduced. It aims to prevent short-lived energy supply shortfalls that occur when the available energy falls below the level required for transmitting critical information. By providing timely and detailed predictions of the future energy situation for a given period, this approach offers a fresh perspective and can significantly enhance the performance of wireless sensor networks [121].

Indeed, achieving high energy efficiency in WSNs involves more than just algorithms. Karchana Kumari has introduced a novel approach by utilizing a new type of cantilever beam that possesses improved mechanical strength and sensing characteristics. This cantilever beam is subjected to a specific amount of strain, and the output voltage of the piezoelectric converter depends on its structure. To optimize the performance of the conventional cantilever beam, Kumari applies the Krill–Herd optimization method. This optimization technique focuses on adjusting both the position of the beam end and its vibration frequency. By employing the Krill–Herd optimization method, the shape and geometry of the cantilever structure within the system are optimized to enhance energy collection effectiveness [122]. By combining innovative material design and structural optimization techniques, Kumari’s work offers a promising approach to improving energy harvesting efficiency in WSNs.

### 5.2. PiENG

In 2016, Geon-Tae Hwang, Venkateswarlu Annapureddy, and their colleagues proposed a novel approach for developing high-performance flexible piezoelectric energy harvesters on plastic substrates. They used the aerosol deposition (AD) method to make piezoelectric films Lead Zirconate Titanate (PZT) to complete the energy harvesting, aiming to enable self-powered wireless sensor node systems. The researchers prepared AD-PZT crystal films with a thickness of 7 μm on a rigid sapphire substrate and subjected them to high-temperature annealing at 900 °C. Subsequently, they successfully transferred these films to an elastic substrate using an inorganic-based laser stripping (IRLO) technique, ensuring that the structural integrity and performance of the films were not compromised. The piezoelectric energy harvesting device developed in this study was subjected to experimental testing, which revealed that it could generate open-circuit voltages of up to 200 V and short-circuit currents of 35 μA through mechanical motion [123]. This research demonstrates the potential of high-performance flexible piezoelectric energy harvesters for powering wireless sensor nodes, offering opportunities for self-sustainability and extended operational lifetimes.

**Figure 11:**
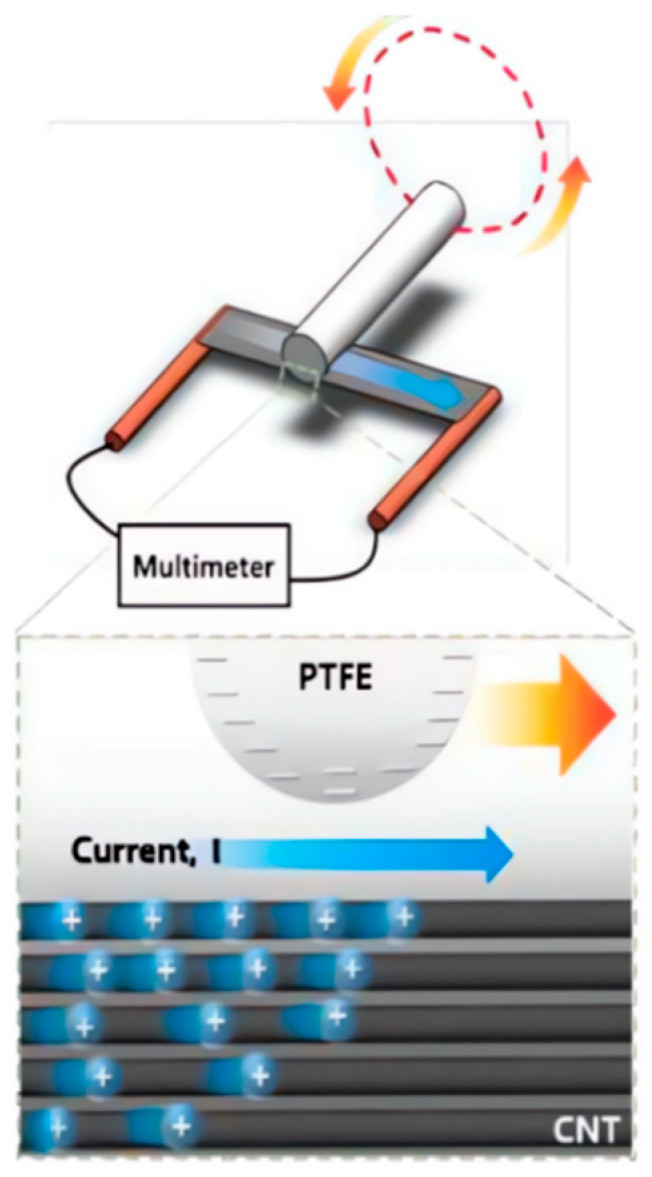
The structure and principle of the nanogenerator [125].

The mechanical energy generated by the vibrations can be collected by means of a piezoelectric device or by using an electromagnetic collector. They can collect enough energy for most applications, but they are not small devices and the vibrations are high and they do not guarantee sustainability, which is not possible on a bicycle movement. Luca Buccolini et al. developed a device to collect electromagnetic energy. This energy-harvesting device consists of a coil that absorbs the energy of the wheel’s rotation and senses the wheel’s rotation. This energy is sufficient to transmit bicycle speed data to a nearby device using a low-power RF protocol. The energy of the collector was tested [124]. As we know, carbon nanotubes have a high surface area relative to their volume and are therefore susceptible to external perturbations. Based on this principle, Hyelynn Song et al. developed a thin carbon nanotube nanogenerator without any fluid or gas flow (as in Figure 11), using the more intuitive coulomb gravity to drag the carriers to generate voltage differences instead of the usual current-induced voltage [125].

### 5.3. PyENG

**Figure 12:**
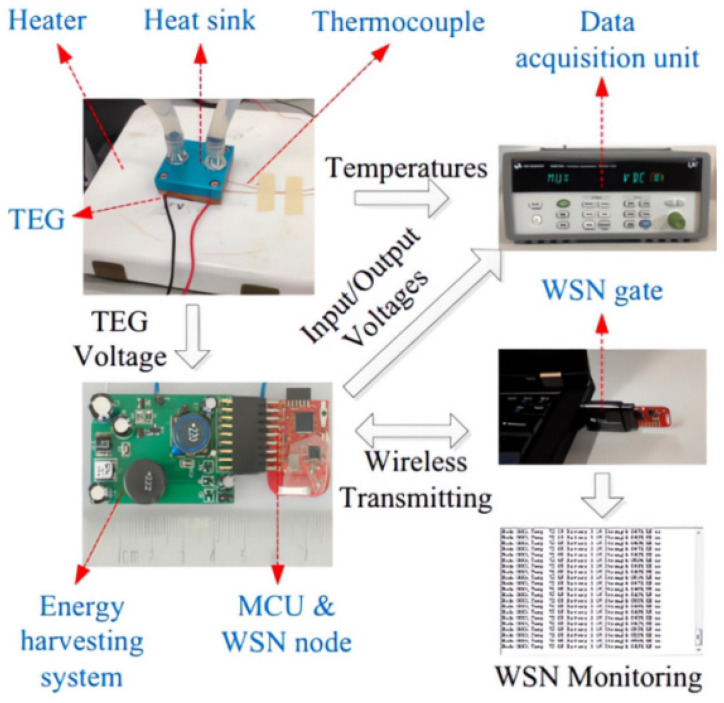
Prototype energy harvesting system and experimental setup [126].

In 2017, Sun Jin Kim, Hyeongdo Choi, and Yongjun Kim focused on addressing the limited output power density of flexible thermoelectric generator (f-TEG) modules due to the low figure of merit (ZT) of screen-printed thermoelectric films. To enhance the ZT value, they employed an ionization defect engineering process and successfully achieved the maximum ZT by utilizing N-type BiTeSe thick films for screen-printing. They developed a new f-TEG device using post-ionization defect engineering (PID) to further increase the ZT of the printed thermoelectric material and improve the output characteristics of the f-TEG module. The energy harvesting device is shown in Figure 12. This research provides a foundation for fabricating f-TEG modules with higher performance and opens up possibilities for more efficient thermoelectric energy harvesting [126].

Body heat has long been recognized as a viable power source for sustainable operation. F-TEG offers a means to collect this body heat. However, traditional inorganic thermoelectric materials are not well-suited for adaptation to human skin due to their rigidity. To address this limitation, there is ongoing research on f-TEG to make them more compatible with human skin. While most f-TEG for body heat harvesting relies on inorganic materials, there have been notable contributions in the field of thermoelectric absorption systems for capturing the heat generated by the physical movement of animals. Smitha Ankanahalli Shankaregowda et al. have published their own work on this topic, highlighting the use of a simple graphite-coated paper (GCP) electrode solution as an efficient and high-quality power source for recycling waste energy. Experimental data demonstrate that the flexible and hydrophobic GCP can effectively absorb waste energy and power wireless sensors [127].

In addition to GCP, PVDF exhibits a strong piezoelectric effect. This polymer material’s pyroelectric effect has attracted significant interest among scientists. Notably, PVDF can be cost-effectively fabricated into thin films and possesses excellent chemical resistance to high electric breakdown fields. Marco et al. explored solution-based transparent electrode (TE) materials, such as silver nanowires (AgNWs) and poly(3,4-vinylenedioxythiophene) polystyrene sulfonate (PEDOT:PSS), for piezoelectric and thermoelectric transparent electrodes in the construction of PVDF wireless sensors. They compared the results with commercially available aluminum alloy electrodes on polymer substrates and highlighted the advantages of these materials. The interaction between piezoelectricity and thermoelectricity shows promise in driving wireless sensor nodes [128].

M. Guan et al. conducted research on the utility model related to a low voltage temperature difference energy acquisition and management system using WSN self-starting. The system is capable of acquiring power ranging from a few tens of microwatts to a few milliwatts from a low-voltage thermoelectric generator, as depicted in Figure 8. In their study, they proposed a new boost converter based on the maximum open circuit voltage tracking technique, employing a bipolar boost mode. Experimental analysis confirmed the suitability of this system for low input voltage and low power operating environments, demonstrating its potential for practical application [129].

**Figure 13:**
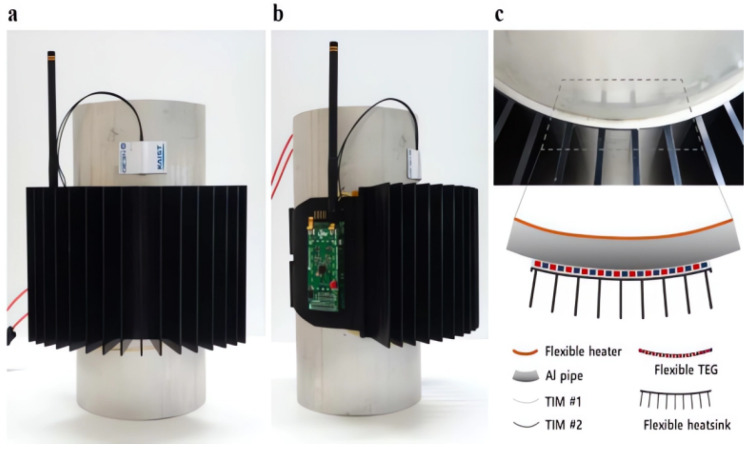
(**a**) Front, (**b**) side, and (**c**) top views of a self-powered WSN driven by a f-TEG. A cross-sectional schematic of the combined heat pipe/f-TEG/heat sink is shown below [130].

In 2018, Kim Yong Jun and Choong Sun Kim aimed to address the limitation of sensor nodes that typically allows only one sensor to operate, which poses challenges in industrial environments where multiple sensors are required. To tackle this issue, they developed a large-area f-TEG that can be wrapped around heat pipes of various diameters, as depicted in Figure 13. This design improvement enhances the usability and scalability of the f-TEG for sensor networks. The primary objective of their solution was to optimize the f-TEG specifically for sensor networks. The developed f-TEG, measuring 140 × 113 mm^2^, achieved an energy output of 272 mW at a temperature of 70 °C from the heat pipe. By utilizing LoRa communication, the system enabled the remote monitoring of heat pipe temperature, ambient temperature, humidity, CO_2_ concentration, and VOC concentration. Additionally, they developed a wireless sensor network (WSN) communication network that allowed wireless data transmission over a distance of 500 m [130]. This advancement in f-TEG technology offers potential benefits in industrial environments by providing a scalable and versatile solution for powering multiple sensors and enabling remote monitoring through wireless communication.

There are several common environmental energy sources, including tidal, thermal, solar, and wind energy. Adnant Jushi et al. have proposed the utilization of wind energy harvesting for passive wireless sensor systems. Their approach involves forecasting weather conditions to anticipate wind energy availability and employing various power manager strategies to efficiently harness wind energy [131].

**Figure 14:**
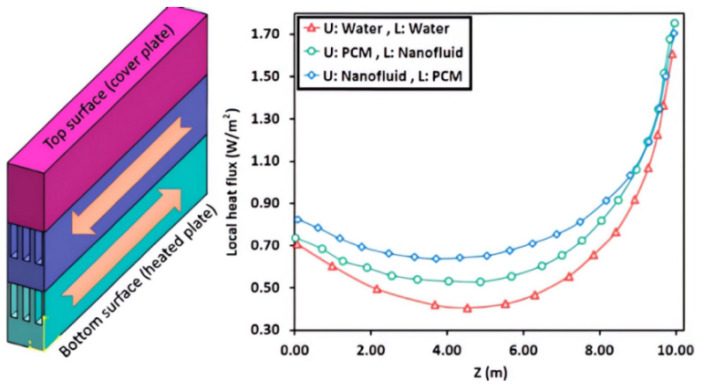
Analysis of a finned heat sink with PCM [132].

In 2019, Park H. et al. presented the application of thermoelectric as a sustainable power source for the Internet of Things, including wireless sensor networks. They addressed the challenge of increasing the low output voltage of thermoelectric systems to more usable levels by integrating power management with DC-DC voltage converters. Furthermore, they proposed improvements to thermoelectric systems, suggesting the use of thermoelectric generators with integrated heat sinks, as depicted in Figure 14 [132].

### 5.4. TENG

**Figure 15:**
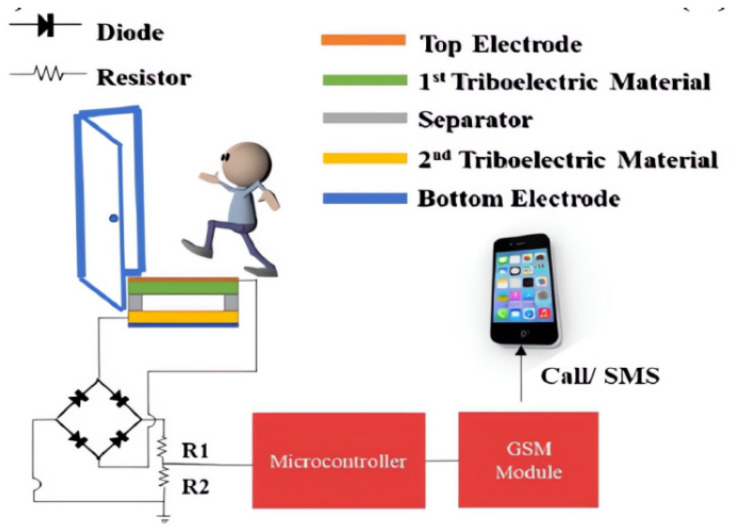
Layout of sensors with self-supplied power [133].

In 2020, Chatterjee, Bhunia et al. reported that the friction generator was constructed with porous PVDF and bacterial cellulose as substrate in the Journal of Materials Chemistry. Their study aimed to control the surface and bulk properties of PVDF films through the process of solution casting and investigate the relationship between these properties and relative humidity. Various humidity conditions were employed during the fabrication of PVDF films, which were subsequently tested independently to determine the optimal conditions for film production. To reduce the reliance on the commonly used, yet toxic, solvent dimethylformamide (DMF), acetone was utilized as the principal solvent in the PVDF film preparation. This approach not only minimized environmental hazards but also provided a means to tune the properties of PVDF films, thereby enhancing the performance of TENG devices. The authors also demonstrate the application of the developed friction nanogenerator self-powered sensor in remote safety. In Figure 15, anyone stepping on a TENG device generates an alternating signal, the size of which is determined by the applied load. The resulting signal is then converted into a DC signal output by a bridge rectifier, and the results demonstrate the high efficiency of this nanogenerator, further highlighting its potential in a variety of applications [133].

In 2022, Alaeldin M. et al. introduced a novel approach for addressing the challenge of the secure detection of passive wireless sensors in railway power supply. Their proposal involved the implementation of a hybrid multi-modal renewable energy harvesting system to support the operation of wireless monitoring sensors, including accelerometers, humidity sensors, tilt sensors, and light sensors, in remote areas along railways. To accomplish this, they developed a hybrid solar photovoltaic system that integrated a 3D-printed wind turbine and an electromagnetic generator. This system effectively harnessed both solar and wind energy, which was then stored in a capacitor for convenient utilization [134].

General nano-energy harvesters typically require a long pre-operation period to accumulate sufficient energy for powering sensors and electronic devices within a short timeframe. This poses challenges for achieving real-time signal transmission, especially in critical scenarios such as emergency and accident monitoring for bridge damage or floods. In 2021, Liangquan Xu and Jinkai Chen proposed a fully self-powered transient wireless sensor system based on a TENG. This system does not rely on electronics or chips but utilizes coils and capacitive passive components. At present, research on WSN based on self-drive mainly focuses on directly converting perceived energy into signals, so there is no need for energy collection, storage, and supply. When the friction nanogenerator is subjected to mechanical vibration, wind, and other external forces, it will be coupled with the resonance loop, thus generating a voltage pulse. This produces a vibrating signal containing an inductive message that can be transmitted wirelessly to a remote receiver for induction. This produces an oscillating signal containing sensing information, which can be wirelessly transmitted to a distant receiver for sensing purposes. To enhance signal amplitude and transmission distance, the TENG system incorporates microswitches that significantly increase the TENG’s output voltage amplitude [135].

**Figure 16:**
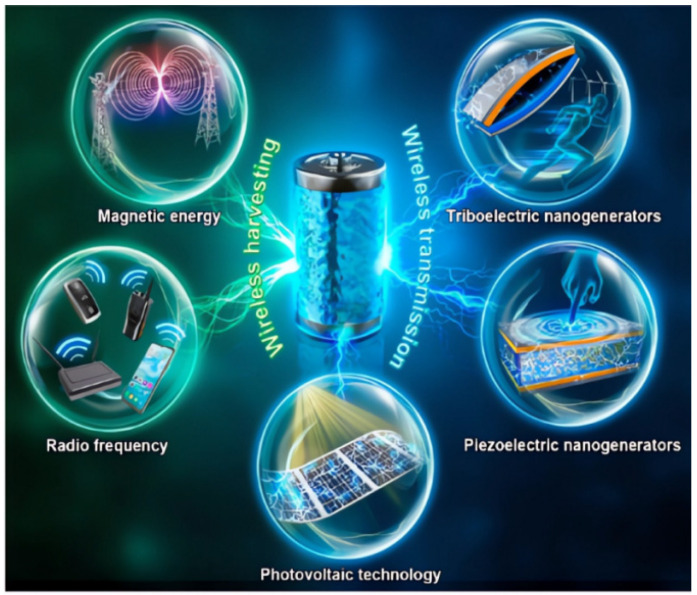
Development of wireless technologies for environmental energy harvesting or transmission [136].

Chengmei Jiang et al. addressed the contemporary challenges in energy harvesting and transmission for enabling the wireless transmission of sensor devices through energy supply. They highlighted the development trends in wireless technology for environmental energy harvesting and transmission, as illustrated in Figure 16. This paper analyzes the various energy sources used in the wireless environment energy acquisition system. On this basis, different acquisition mechanisms and wireless transmission modes in energy acquisition and wireless transmission systems are studied [136]. The authors also propose finding an effective method for fabricating a small and easily integrated sensor node power supply device with high harvesting efficiency, high power density, and wide bandwidth and spatial coverage, as well as easy installation and maintenance.

Current challenges regarding energy harvesting have the following factors. First, randomness is the biggest limiting factor for external energy. When the external energy source changes, the sensor is bound to be affected. For example, when the external temperature difference cannot reach the minimum set temperature difference of the thermoelectric nanomaterials, the thermoelectric effect cannot be carried out. When external mechanical energy cannot be guaranteed, piezoelectric materials cannot absorb energy. Secondly, energy substitution efficiency is also a major limiting factor for wireless passive sensors. Although the sensor has low power consumption, it will continuously lose energy during transmission, and it will not be able to maintain the stable operation of the wireless sensor if the conversion efficiency is not guaranteed. Moreover, when such sensors are applied to real life, the size must also be considered, such as in implanted human health detection and structural detection, when too large a volume is not allowed. So, size is also a limiting factor. Finally, there is the requirement for the speed of energy collection, because, in many cases, the energy storage device does not have a working space, so it needs to supply energy directly. At this time, there are higher requirements for the speed of energy absorption. How to reduce the impact of these limitations on our future applications is the most critical question. Miniature nanogenerators have become integral components of passive wireless sensors (as listed in Table 3), offering a new and environmentally advantageous method for electricity generation. Nanogeneration, as a technology, presents several benefits, including simplified energy loading and unloading processes. With ongoing advancements in microdevices, the expansion of nano-powered technologies, particularly in wireless sensing, is inevitable and holds promising application prospects. Table 4 provides a comprehensive summary of the three main types of nanogenerators and highlights the distinctions between the various energy sources utilized.

## 6. Passive Wireless Sensor Applications

In recent years, significant progress has been made in the development of passive wireless sensor production processes and continuous innovation. These advancements have resulted in new breakthroughs in passive wireless sensor devices, leading to more precise parameter readings. There is a growing emphasis on translating theoretical concepts into practical applications to serve the public better. Notable examples of these applications include building structural safety testing, where passive wireless sensors play a crucial role in monitoring and ensuring the integrity of structures. Additionally, these sensors are employed in monitoring human health, facilitating the continuous detection and early identification of health-related issues. Moreover, sensors contribute to the safety testing of commonly used equipment, ensuring efficient and reliable operation.

The drive to bridge the gap between theory and application is motivated by the desire to enhance safety and well-being. Wireless sensors have the potential to anticipate safety hazards in advance, enabling proactive measures to be taken promptly. By offering real-time monitoring and analysis, these sensors empower individuals and organizations to implement timely protective actions. The integration of passive wireless sensor technology brings significant benefits to various fields, enabling safer building structures, improved healthcare monitoring, and enhanced equipment safety. The focus on practical implementations reflects the commitment to better serve the public and address their evolving needs. Overall, the advancements in passive wireless sensor devices and the translation of theoretical knowledge into practical applications are instrumental in delivering tangible benefits to society, fostering safety, and enriching people’s lives.

### 6.1. Structural Safety Testing

Industrial progress has significantly enhanced quality of life, leading to an increased emphasis on safety. Consequently, products with high levels of security have become the preferred choice. Wireless sensors play a crucial role in improving safety by anticipating potential hazards and enabling prompt preventive measures.

Soil erosion poses substantial risks such as house collapse and safety accidents caused by unstable soil alongside roads. In 2010, Bogdan Lie and Kenneth J. Loh proposed a passive wireless sensor design for soil structure detection. This design incorporates epoxy resin encapsulated artificial soil particles to detect displacement and identify the likelihood of soil loosening. By embedding these particles in the soil, their movement at the soil-structure interface can be monitored, providing insights into the mechanics and behavior of the soil-structure interface [137].

SHM is vital for detecting small deformations and cracks in structures. In 2016, Chunhee Cho et al. developed a passive wireless sensor for precisely detecting such deformations and cracks. This sensor employs a phototransistor modulated antenna sensor due to the low sensitivity of conventional RFID antenna sensors to strain. The system detects variations in resonance frequency generated by the patch antenna’s length. Additionally, a frequency doubling mode is utilized to distinguish ambient scattering signals from sensor scattering signals, enabling accurate micro-strain measurements. The sensor exhibits a strain sensitivity approximately five times higher than previously developed RFID antenna sensors [138].

Infrastructure has a limited lifespan, and regular maintenance checks become necessary. Traditional visual inspections often fail to detect issues promptly and require skilled personnel and significant time investment. Wireless sensor inspections offer a convenient solution to this challenge. In 2019, E. Matsunaga et al. designed a passive wireless sensor for efficient remote inspections [139]. The sensor’s resonance frequency change caused by the varying resonant length is utilized to determine if a bolt is loose, and the feasibility of the approach was demonstrated through experiments.

Metal structures are susceptible to oxidation and corrosion, making safety inspection of paramount importance. However, certain metal structures are difficult to access for inspection, such as those submerged in seawater. In 2016, Rania Khalifeh et al. proposed a wireless passive corrosion sensor that employs chipless RFID technology. This sensor enables the monitoring of degraded submersible infrastructure located in coastal areas and submerged environments. The sensor can be deployed in areas submerged in seawater or splash zones. By modifying the potential of the sensitive metal and detecting changes using two resonators integrated with an antenna, corrosion can be effectively detected. Experimental results validate the suitability of this method for subsea monitoring [140].

To meet the demands of smart city construction, self-propelled devices offer innovative approaches for traffic monitoring in intelligent cities. This encompasses fire alarms, environmental monitoring, aircraft crack detection, sensing hazardous chemicals in high-traffic areas, and monitoring vehicle tire stress, temperature, and pressure. Hassan Askari and Ehsan Asadi proposed a hybrid electromagnetic-friction generator that can power road monitoring sensors, facilitating traffic monitoring. This hybrid approach capitalizes on the benefits of hybrid design, providing novel ideas for future nanogenerator development [141].

For structural health testing, applications in vehicles are essential. Generally, the vehicle health detection we use is judged by the driving distance and inspection cycle, which obviously has very low reliability [142]. The development of passive wireless sensors can realize the real-time detection of vehicles, through the detection of vehicle battery consumption, traffic distance, and other parameters and avoid driving hazards due to battery damage [143].

### 6.2. Human Health Testing

In the realm of human health detection, wireless sensors play a crucial role in generating electrical signals and collecting information for assessing human well-being. The real-time monitoring of physiological signals, such as implanting organs for disease treatment and integrating systems for remote monitoring, can significantly reduce medical stress and provide individuals with real-time health data.

The clinical management of traumatic wounds still largely relies on traditional approaches from the 20th century. Wound trauma assessment is primarily based on visual observation by physicians, often leading to frequent dressing modifications. However, the excessive use of wound dressing changes disrupts the healing process and escalates the cost of treatment. Visual inspection, being subjective, may not always provide an accurate representation of the wound, resulting in the risk of unnecessary replacements. Detecting inappropriate wounds accurately would enable appropriate treatment by medical personnel, preventing severe wounds from becoming chronic. Rahimi, Brener, and Ochoa et al. implemented a flexible pH sensor connected to an RFID transponder with a wireless module that enables wireless pH readings via an NFC-enabled smartphone. This approach facilitates visual inspection, and the sensing electrode, fabricated using low-cost ITO film directly laser scribed, offers practicality for real-life applications [144].

**Figure 17:**
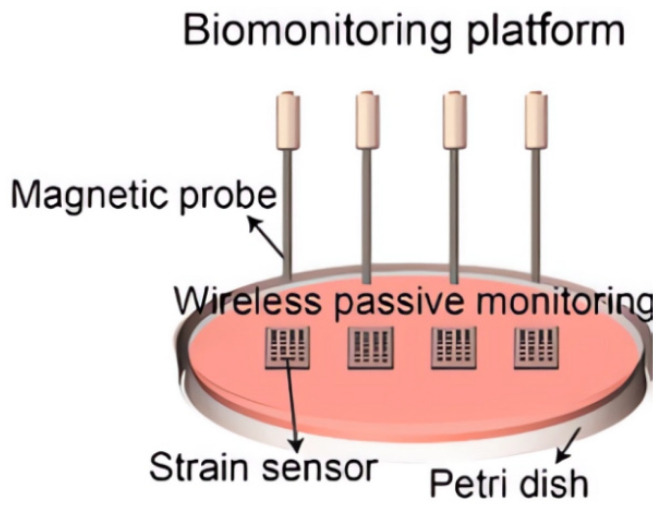
Bio-detection using wireless passive sensors [148].

In addition to PH, mechanical stress and moisture content in skin tissue significantly influence the rate and efficacy of wound repair and necessitate real-time monitoring. Studies have demonstrated that moderate wound pressure facilitates the formation and growth of new blood vessels, while maintaining moisture balance promotes growth and migration, essential for optimal wound repair. The real-time monitoring of water pressure and humidity surrounding the wound is crucial for assessing the environmental conditions conducive to wound healing and improving patient comfort. In 2018, Wang Lifeng and Huang Qingan designed flexible passive wireless pressure and humidity sensors by utilizing a moisture-sensitive capacitor with GO as the sensing material and a pressure-sensitive capacitor with conical PDMS as the medium. The flexible nature of the sensor materials enhances their suitability for wound management. The real-time wireless monitoring of mechanical pressure and humidity at the wound site, along with the prediction of optimal wound healing time, was achieved. The wireless sensor exhibited a humidity sensitivity of −61 kHz/%RH in the range of 20% RH to 90% RH and a pressure sensitivity of −388.6 kHz/mmHg in the range of 0 mmHg to 100 mmHg [145]. In 2021, Sadaf Charkhabi et al. proposed a wireless sensor for monitoring wound healing. The sensor tracks the healing process while providing a hygienic system for the wound, ensuring a sterile environment. The sensor determines specific parameters based on the effect of the healing degree on the dielectric constant and electrical conductivity of the tissue. Structural modeling tests on gelatin validated the effectiveness of the sensor, representing a novel breakthrough in human health monitoring [146]. Particularly in cases like the COVID-19 pandemic, where many healthcare workers are unable to personally monitor the health status of patients, wireless sensors offer significant alleviation of this stress. Nappi, Miozzi et al. proposed a human skin patch utilizing RF identification technology to monitor various skin parameters such as temperature, humidity, etc. The sensor was tested considering the degree of harm to the human body, and the results demonstrated its ability to read data up to 80 cm, thereby enhancing healthcare practices [147]. In 2022, Qi Zhang et al. utilized flexible sensors to enable the real-time monitoring of human health and disease determination through passive wireless sensors. Sensors fabricated from Fe3O4 magnetic hydrogels exhibit remarkable mechanical properties, characterized by their ultra-softness, and possess a powerful magnetic force. These unique attributes enable more accurate monitoring of strain levels, as demonstrated in Figure 17, representing a significant advancement in the field of implantable wireless passive sensors for human body applications [148].

In addition to monitoring traumatic wounds, wireless sensors also play a significant role in monitoring various other health conditions, reducing hospital visits, lowering costs, and providing accurate health information. Glaucoma, the second leading cause of blindness worldwide, affects a large number of individuals each year, leading to vision loss. It is an eye disease associated with high intraocular pressure (IOP). Currently, glaucoma patients require the close monitoring of their IOP, which is managed through surgical procedures and medications. With the improvement in living standards, the prevalence of vision problems is increasing. Monitoring IOP parameters can serve as an important indicator of eye health.

M. Hossein M et al. developed a passive wireless sensor embedded in a contact lens to enable the real-time monitoring of IOP. This innovative approach allows the continuous monitoring of corneal changes resulting from IOP fluctuations. The sensor primarily consists of a constant capacitor and a variable inductor. Notably, the inductor’s shape is a closed-loop serpentine. This technology offers a significant advancement in monitoring and managing glaucoma [149].

In the field of bone performance assessment, Limin Ren and his student Kun Yu proposed a magnetoelasticity-based artificial bone force monitoring sensor. This sensor aims to enhance non-invasive methods for evaluating bone performance and provides timely feedback for postoperative management. By utilizing this sensor, healthcare professionals can monitor bone forces and optimize patient care accordingly [150].

Parkinson’s disease is a progressive neurodegenerative disorder characterized primarily by tremors in the hands, feet, or body, along with a range of other symptoms including bradykinesia (slow movements), rigidity, facial immobility, reduced arm swing during walking, and speech difficulties such as reduced volume, monotony, and slurring. To effectively monitor patients with Parkinson’s disease, Jong-nam Kim et al. have developed a self-powered tremor sensor using catechol chitosan diatom hydrogel (CCDHG), a highly ductile and ionically conductive material. CCDHG is composed of naturally abundant marine biomaterials that possess self-healing properties and conductivity even under repeated high strain conditions. This polymer exhibits excellent flexibility, allowing easy integration with flexible electronic components. By analyzing the CCDHG-TENG in the frequency domain, as demonstrated in Figure 11, the severity of Parkinson’s disease can be assessed without the need for commercially available acceleration sensors. The outcomes of this research project not only offer innovative insights for clinical applications but also provide valuable technical tools to aid in the management of Parkinson’s disease.

Cardiovascular disease has emerged as the most prevalent and devastating condition worldwide, accounting for 12.3 million deaths in 1990 and rising to 17.3 million deaths in 2013. Extensive research indicates that the long-term monitoring of physiological signals can predict approximately 90% of cardiovascular diseases. To address this, Han Ouyang et al. have devised an innovative and flexible pulsed sensor based on frictional electro-active sensors. This self-driven sensor exhibits remarkable features such as high sensitivity, a high peak signal-to-noise ratio, and low cost. The system enables the precise, wireless, and real-time monitoring of the cardiovascular system using analyzing pulsed signals. The analysis includes pulse profiling, HRV pangaratograms, and Time HRV indices, as well as the identification of coronary heart disease, septal defects and atrial fibrillation [151].

Thermal energy, which is abundantly present in various natural and human-related sources, such as solar thermal energy, vehicle exhaust emissions, and human respiration, can be effectively harnessed. PyNGs offer a promising solution for converting waste heat into usable energy. In this regard, Hao Xue and their team have developed wearable PyNGs by integrating a PVDF membrane with an N95 mask. This innovative device captures the heat generated during human respiration, enabling the real-time monitoring of breathing patterns and the surrounding temperature [152].

The development of wireless passive sensors provides convenience for the use of the Internet of Things [153]. With the enhancement of social health awareness, students’ physical and mental health has been widely concerned. But many factors determine physical and mental health, and at this time, the Internet of Things and wireless sensors have become a promising and effective solution. Li Hong-tan et al. proposed the use of wireless sensors to capture students’ external environmental factors and collect various indexes that affect students’ happiness in daily life, such as body BMI, students’ examination scores, and daily exercise frequency. Then, the Internet of Things is used to collect data, and various artificial intelligence algorithms are used to judge the physical and mental health of students. It has realized the monitoring and management of the healthy development of adolescents [154].

### 6.3. Safety Testing of Commonly Used Equipment

In our daily lives, ensuring the safety of machinery and equipment is of paramount importance. However, monitoring these devices can pose challenges due to the harsh environments they operate in. This is where wireless sensors can prove invaluable.

**Figure 18:**
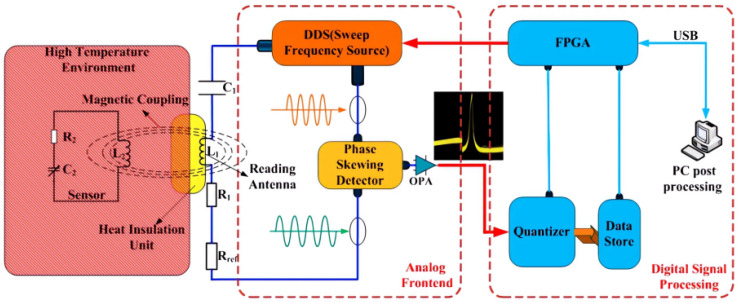
Block diagram of a wireless measurement system [155].

In 2015, Huixin Zhang and Yingping Hong developed a set of passive pressure measuring systems suitable for high temperature environments above 800 °C. The system uses a ceramic-based liquid crystal resonance sensor, an impedance phase detector reader, an insulator, and a composite temperature and pressure test platform, as shown in Figure 18 [155]. The experimental results show that the linearity of the sensor is 0.93%, the repeatability is 6.6%, the hysteresis error is 1.67%, and the sensitivity is 374 kHz/bar. It can accurately measure temperatures from room temperature to 800 degrees Celsius and pressures from 70 to 190 degrees Celsius. Notably, the sensor’s capabilities make it suitable for measuring the internal chamber pressure of fuel vehicles.

In 2020, Zhu Jianhua et al. made use of passive wireless sensors to detect temperature changes in transformers, presenting a new approach to wireless temperature measurement. This innovative wireless device offers improved safety measures for transformers by effectively monitoring and maintaining stable working temperatures [156].

In the field of drilling, the accurate measurement of drilling speed and orientation is crucial for regulating the drilling process. Zhou et al. have developed a new type of sensor that enables precise measurement of drilling speed and orientation with an error of 4%, a sensitivity of 0.0167 Hz/rpm, and a linearity of 3.5%. This sensor, with a range of 0–1000 rpm, helps avoid delays and economic losses by eliminating the need to lift the drill bit from the well when the speed sensor is replaced or damaged [157].

Another noteworthy development comes from Lifeng Zhu, who has created a self-powered rotational motion sensor capable of measuring multiple parameters simultaneously. The sensor incorporates friction electrodes, primarily consisting of rack-shaped plates and incomplete gears, which enhance the output signal. Additionally, the sensor demonstrates resistance to environmental noise, making it suitable for industrial applications in the field of rotary detection [158].

In the realm of self-powered sensors, Di Yu et al. have produced a PiENG based on (BaTi_0.88_Sn_0.12_O_3_) BTS, a lead-free piezoelectric ceramic doped with tin BaTiO_3_ (BTS). These flexible composite films can undergo significant changes in response to small force variations. This self-powered sensor, utilizing the BTS-GFF/PVDF composite film, is suitable for monitoring human motion and fatigue driving safety, which is particularly important in addressing the global issue of traffic accidents [159].

For the safe operation of railway systems, Xuejun Zhao and Guo Wu propose a frictional electro-nano-vibration velocity sensor with self-driven characteristics. This sensor effectively collects and converts low-frequency vibration energy and can serve as a self-driven vibration acceleration sensor. These research findings lay the foundation for the application of Railway Structural Health Monitoring technology in railway locomotive equipment and practical operations [160].

Tyre pressure monitoring is crucial for ensuring the safety and effective use of tyres in the automotive industry. Jingui Qian and Dong-Su Kim have developed a magnetic-accelerated sensor and a magneto-frictional nanogenerator that can operate a wireless sensor for the real-time transmission of tyre pressure and temperature data. This technology reduces the risk of equipment failure caused by excessive centrifugal forces, friction, and jamming, thus improving the safety and reliability of tyre monitoring systems [161].

In the field of earthquake monitoring, Limin Ren proposes a self-supplied magnetostrictive sensor that utilizes Faraday’s induction principle. The sensor detects changes in magnetic flux caused by variations in magnetic permeability and generates a voltage across a coil to alert of an impending earthquake. This technology holds practical significance in safeguarding human lives and property by enabling effective earthquake monitoring [162].

## 7. Summary and Outlook

In this paper, the passive wireless is classified into an RFID sensor, a harmonic scattering sensor, a passive resonance sensor, and a self-powered sensor according to the different working modes of the sensor. A brief description of how these sensors work is followed by a list of the types of sensitive materials prepared for testing sensors with different parameters. Several methods to improve the efficiency of sensor communication are illustrated. The development of energy harvesting nanomaterials in nanogenerators is pointed out, and the application of these sensors in daily life is introduced. Passive wireless sensors have been a research hotspot in recent years, and these types of sensors have their advantages.

With the continuous progress of science and technology, passive wireless sensors are playing a crucial role in numerous applications and attracting the attention of researchers from various fields. The requirements for the use of sensors are also increasing, such as the quality factor, sensitivity, and the use space of the sensor. Innovations in sensitive materials such as semiconductors and polymer materials will drive the development of sensors. These sensors are utilized for measuring parameters such as pressure, humidity, strain, temperature, and pH values. Due to their unique characteristics, wireless sensors can be employed to monitor locations that are difficult to access directly, thus playing a significant role in everyday life. Operating at low frequencies, typically in the tens of megahertz range, and utilizing near-field coupling technology, they enable short-range data transmission and energy transfer in harsh environments without the need for active equipment such as batteries. They are simple to operate, have low maintenance costs, and are environmentally friendly.

Furthermore, in terms of parameter reading, there are limitations such as large data reading errors, limited reading distances, and the inconvenience and limited availability of network analyzers in the actual use of passive wireless sensor systems. When designing automatic and portable impedance matching systems, effective solutions need to be proposed to enhance the usability of passive wireless sensors, such as extending the reading distance and improving reading accuracy. In terms of data transmission, because passive wireless sensors often rely on low-power communication strategies for transmission, they often lead to problems such as low transmission rate and limited transmission distance. Therefore, it is also a problem to improve the transmission efficiency while meeting the sensor power consumption. Regarding energy harvesting, various methods exist to collect environmental energy from radio waves. Future research should focus on exploring wireless sensor network topologies and developing new algorithms to reduce energy losses and minimize the requirements for nano-acquisition devices. At present, the efficiency of energy recovery during the collection process is still not high. Additionally, wireless sensors are susceptible to interference from the surrounding environment, such as metal and liquid, and the collection efficiency of low RF power density and low-frequency magnetic field environments is relatively challenging to meet the energy requirements. The continued exploration of the applications of wireless sensors in daily life and the expansion of their usage to serve the public more conveniently is an important area for future development. Such sensors are often used in environments that require long-term work, so they also face problems such as energy attenuation and equipment aging. With prolonged use time, the efficiency of the sensor may decrease, the drift phenomenon becomes serious, and eventually, it cannot operate. Therefore, it is also urgent to explore new materials and research new manufacturing technologies.

## Figures and Tables

**Table 1:** Summary of sensitive materials for various types of sensors.

Sensor Type	Sensitive Materials	Uses	Range of Tests	Sensitivity	Operating Frequency	Others
Temperature sensors [57]	Epoxy	Real-time detection of high-voltage safety circuit systems	200 to 1200 °C	7.016 kHz/°C	428–439 MHz	Operating frequency: 1.7 °C/s
Temperature sensors [58]	Alumina ceramic	High-voltage safety circuit systems	25 to 1000 °C	2 kHz/°C	27.6 MHz	Sensor quality factor: 78
Temperature sensors [61]	Nickel, Silicon	Food quality testing	0 to 100 °C	46.61 kHz/°C	Around 137.00 MHz	-
Temperature sensors [64]	SiCNO-BN	High temperature testing	Up to 1250 °C	-	10.706–10.693 GHz	-
Temperature sensors [65]	Ceramic Oxide	Real-time detection of high-voltage safety circuit systems	200 to 1200 °C	170 kHz/°C	Around 50 MHz	Sensor quality factor: 34.5–43
Humidity sensors [68]	Polyimide, Ag	-	15~95% RH	−3.7%	13.56 MHz	-
Humidity sensors [69]	Graphene oxide	-	15~95% RH	18.75%	30–40 MHz	-
Humidity sensors [70]	Synthesis of silicon dioxide with hydrophilic alkene-based monomers	-	11~95% RH	40.1%		Operating frequency: 3.58 RH/s
Humidity sensors [72]	Optimisation of graphene oxide	-	Best 32.8% RH	40.1%	-	5 RH/s
Pressure sensors [74]	Polyimide, acrylic, Cu	-	0–60 mmHg	3 kHz/Pa	109 MHz	-
Pressure sensors [77]	Sapphire	-	Up to 800 Pa	21.7 kHz/Pa	12.4–18.0 GHz	-
Pressure sensors [81]	Optimisation of graphene oxide	-	17–200 kPa	1.5 ppm/kPa	886.7 MHz	Response time: 1 s
Gas sensors [85]	P(VDF-HFP) polymer	Carbon dioxide detection	5–10 ppm	-	11.976 kHz	-
Gas sensors [87]	SnS2, Ceramic		77–1155 ppm		21.42 MHz	Dimensions: 25.22 × 0.98 mm^3^
Gas sensors [88]	ZFO-NF	Hydrogen Sulphide Detection	0–1 ppm	-	-	Response time: 10 s
Gas sensors [89]	Graphene oxide and niobium-doped titanium dioxide	Hydrogen detection	-	-	-	-
Biosensors [91]	Phenylboronic acid-hydrogel	Glucose	-	304 KHz/(mg/dL) -	Around 1 GHz	Size: 5 mm × 5 mm × 250 μm
Biosensors [92]	Hot gelatine—glycerine—ca composite	Protein	-	-	-	-

**Table 2:** Summary of Passive Wireless Sensor Types.

Type	Advantages	Disadvantages	Applications
RF Identification (RFID) [97]	Low power consumption	distances sensor node circuit is complex	Positioning, tracking
Surface Acoustic Wave (SAW) RFID [100,101]	High sensitivity	High cost and power consumption	Measuring temperature, pressure, velocity of flow
Harmonic sensors [103,107]	High sensitivity, low false alarm rate	Demanding for readout systems	Positioning
Capacitive inductance (LC) mode of resonance [95,109]	Simple structure, low power consumption	Vulnerable to external electromagnetic interference	Measure temperature, pressure, humidity
Radio frequency (RF) cavity type [94]	Long transmission distance	High working frequency band, large volume	Measuring liquid and gas concentrations
Patch Antenna type [102]	Small size, low cost	The working frequency band is high and will be affected by occlusions	Human posture detection

**Table 3:** Summary of each type of nanogenerator.

Type	Advantages	Disadvantages	Applications
PiENG [123,125]	High energy collection, high sensitivity, low frequency operating range	Piezoelectric materials are often toxic Fragile	Powering wireless sensor arrays
PyENG [127,129]	Easy to implement collection environment	Low power collection	Wireless charging for phones, powered by sensor arrays
TENG [133]	Self-starting without external force	Difficult to maintain the tightness of the friction material	Powering LEDs, small microcontrollers

**Table 4:** Summary of energy differences.

Type	Advantages	Disadvantages	Applications
Light Energy [121,134]	Large energy supply	Higher costs	Wearable wireless monitoring system
Mechanical energy [124]	Cleaning	High environmental requirements	Passive wireless SAW pressure sensors
Thermal Energy [127,129]	Convenient collection conditions	Low efficiency	Wireless charging for phones
Chemical energy [136]	Achieving controlled conditions	High degree of restricted use	Wireless Communication, Passive Wireless Biosensors
Human Movement Energy [133]	Convenient collection conditions	Collecting less energy	Sound judgement

## Data Availability

Not applicable.
